# Identification of Factors Influencing Variability in Disinfection Byproducts and Their Toxicity in Chlorinated and Chloraminated Drinking Water Distribution Systems across the United States

**DOI:** 10.1021/acs.est.5c12121

**Published:** 2025-12-31

**Authors:** Samantha DiLoreto, Huanqi He, Jinhao Yang, Patrick Milne, Jiaqi Li, Christopher A. Impellitteri, Aron Stubbins, Ameet Pinto, Ching-Hua Huang

**Affiliations:** † School of Civil and Environmental Engineering, 1372 Georgia Institute of Technology, Atlanta, Georgia 30332, United States; ‡ School of Science and Engineering, Benedict College, Columbia, South Carolina 29204, United States; § Department of Chemistry and Chemical Biology, 1848 Northeastern University, Boston, Massachusetts 02115, United States; ∥ The Water Tower, Buford, Georgia 30519, United States; ⊥ Department of Marine and Environmental Sciences, Northeastern University, Boston, Massachusetts 02115, United States; # Department of Civil and Environmental Engineering, Northeastern University, Boston, Massachusetts 02115, United States; ∇ School of Earth and Atmospheric Sciences, Georgia Institute of Technology, Atlanta, Georgia 30332, United States

**Keywords:** United States, regulated disinfection byproducts, nonregulated DBPs, drinking water distribution systems, cytotoxicity, genotoxicity, Spearman correlation, redundancy discriminant analysis, random forest regression

## Abstract

Drinking water distribution systems (DWDSs) are dynamic environments where disinfection byproducts (DBPs) form and transform, shaped by factors such as the disinfectant type, water chemistry, biofilms, and pipe materials. Understanding the occurrence and drivers of DBP formation within DWDSs is essential for evaluating water quality and potential health risks. This study examined DBP occurrence and variability across eight utilities in the United States, three using chlorination and five using chloramination (as secondary/residual disinfectants), by analyzing 152 quarterly samples collected from multiple locations within each distribution system. Regulated DBPs were found at the highest concentrations. Haloacetonitriles (HANs) and haloacetic acids (HAAs) contributed most to the predicted toxicity. Spearman correlations, redundancy discriminant analysis, and random forest regression revealed how key influencing predictors of DBPs vary between chlorinated and chloraminated systems. Elevated dissolved organic carbon (DOC) and primary disinfectant type were the two most important contributors to DBP formation, with DOC most influential in chloraminated systems and primary disinfectant type (HOCl vs O_3_) in chlorinated systems. Variations in DBP concentrations within individual DWDS showed a weak dependence on water age. This study provides a novel data set linking DBP occurrence to water quality parameters and demonstrates how such data can be leveraged to understand DBP dynamics and inform future risk management in water distribution systems.

## Graphical abstract

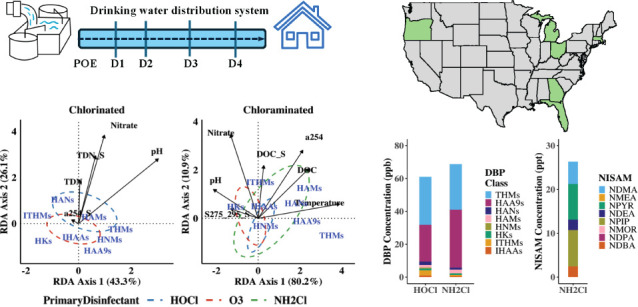

## Introduction

Disinfecting drinking water is essential for removing pathogens, but disinfectants like chlorine can react with natural organic matter (NOM) and inorganics such as bromide and iodide to form disinfection byproducts (DBPs).^1^ Since the discovery of trihalomethanes (THMs) in 1974, over 800 DBPs have been identified, yet they account for only 30% of total organic halogens (TOX), suggesting that many DBPs remain unknown.^2,3^ Some identified DBPs are cytotoxic, genotoxic, or developmentally toxic. Despite these risks, only a few DBPs are regulated by the U.S. Environmental Protection Agency (EPA), including four THMs, chloroform (TCM), bromodichloromethane (BDCM), dibromochloromethane (DBCM), and bromoform (TBM), and five haloacetic acids (HAAs), chloroacetic acid (MCAA), bromoacetic acid (MBAA), dichloroacetic acid (DCAA), dibromoacetic acid (DBAA), and trichloroacetic acid (TCAA).^2,4^

To meet regulations, many utilities have switched from chlorine to alternatives like chloramine or ozone, which reduce regulated DBPs but may increase more toxic unregulated ones, like nitrogenous (N-DBPs) and iodinated DBPs (I-DBPs).^5–9^ For instance, chloramine poorly oxidizes hypoiodous acid (HOI), allowing I-DBP formation. Chloramination also raises N-DBP levels,^6,10,11^ and preozonation before chloramination can increase halonitromethanes (HNMs).^5,10^ Toxicity-based methods now help identify which DBP classes contribute most to overall risk, supporting traditional cell-based assays.^12^ Ongoing monitoring and toxicity assessments of unregulated DBPs remain critical for public health.

Understanding the formation and behavior of unregulated DBPs in drinking water distribution systems (DWDSs) is critical due to potential health risks. While much research has focused on tap or finished water, recent studies have emphasized DBP dynamics within DWDSs.^13–15^ The dynamics of DBPs vary widely across systems because DBP transformations are influenced by factors such as source water, treatment processes, primary and residual disinfectant types, pipe materials, biofilms, residence time, and inorganic and organic precursors.^16^ However, past studies have been limited by a narrow DBP focus, few systems or sampling sites, short sampling periods, and incomplete water quality data. Although microbially derived organic matter is a known precursor for DBPs,^17^ few studies have linked DBP concentrations to microbial cell counts, limiting understanding of the trade-off between pathogen control and DBP formation. These gaps highlight the need for a more comprehensive investigation into the occurrence and behavior of DBPs within DWDSs.

In this study, we aimed to assess how DBP speciation is influenced by disinfection scheme, organic matter content, temperature, and other parameters in DWDS samples across eight utilities in the U.S. These utilities represent a broad range of drinking water and treatment characteristics, enabling a comprehensive assessment. The objectives were to 1) understand the occurrence and formation of 45 regulated and unregulated DBPs in distribution system (DS) and point of entry (POE) samples of DWDSs; 2) calculate cytotoxicity and genotoxicity for individual DBPs and determine which DBPs are the main drivers of toxicity; 3) investigate relationships between various water quality parameters and organic matter content to determine which are influencing DBP formation; and 4) assess the role of engineered factors in DBP formation. This study differs from previous work in that it examines a broad suite of DBPs across multiple treatment processes and source water types, integrates toxicity data, and applies complementary statistical and machine learning approaches to identify key formation drivers in real-world DWDSs.

## Methods

### Chemicals and Reagents

The names, abbreviations, and sources of standards for the target DBPs, surrogates, and internal standards are listed in Supporting Information (SI) Table S1. The DBPs included four THMs, nine HAAs, seven haloacetonitriles (HANs), four haloacetamides (HAMs), trichloronitromethane (TCNM), three haloketones (HKs), six iodinated THMs (I-THMs), two iodinated HAAs (I-HAAs), and nine nitrosamines (NISAMs). Ascorbic acid from Fisher Scientific was used as an oxidant-quenching agent. Other reagents (methyl *tert*-butyl ether (MTBE), acetone, methanol, and LC-MS grade water) were analytical grade and purchased from either Sigma-Aldrich or Fisher Scientific. Ultrahigh-purity nitrogen was purchased from Air Gas. Reagent-grade water (18.20 MΩ·cm) was produced by a Barnstead GenPure Pro water purification system.

### Sample Location and Collection

Samples were collected from eight drinking water treatment utilities (termed U2–U9) across the U.S. from January to December 2024. U2, U6, and U8 use disinfection schemes (primary/secondary disinfectants) of ozone (O_3_)/chlorine (HOCl), O_3_/HOCl, and HOCl/HOCl, respectively, and are considered chlorinated systems in this study. U3, U4, U5, U7, and U9 use O_3_/chloramine (NH_2_Cl), O_3_/NH_2_Cl, HOCl/NH_2_Cl, HOCl/NH_2_Cl, and NH_2_Cl/NH_2_Cl, respectively, and are considered chloraminated systems in this study. See Table S2 for more utility information.

Water samples were collected at each utility before treatment (source water), after treatment (point of entry (POE)), and in the distribution system (DS). The number of samples varied by utility, and water age varied by and within utilities depending on the sampling site locations within the distribution system. Sampling occurred in four rounds throughout the year (details in Text SA1). Samples were shipped on ice (4 °C) to the Georgia Institute of Technology (Atlanta, Georgia, United States) for DBP analysis. Samples for microbial analyses and ammonia were also shipped to the Georgia Institute of Technology (Atlanta, Georgia, United States). Additional samples were sent to Northeastern University (Boston, Massachusetts, United States) for analysis of dissolved organic carbon (DOC), absorbance at UV 254 nm, total dissolved nitrogen (TDN), and other organic matter parameters. Samples for anion analysis were sent to The Water Tower (Buford, Georgia, United States).

### Quantification of DBPs and Water Quality Parameters

Details of the following analytical methods and their quality assurance and quality control (QA/QC) are included in Text SA2. Briefly, THMs, I-THMs, HKs, HANs, TCNM, and HAMs were extracted from water by liquid–liquid extraction (LLE) with MTBE and analyzed using gas chromatography coupled with electron capture detection (GC-ECD, 7890A GC System, Agilent Technologies, United States), following the EPA Method 551 with modifications as described in Text SA2. HAAs and I-HAAs were extracted using LLE with MTBE followed by acid derivatization and analyzed by GC-ECD, following the EPA Method 552 with modifications as described in Text SA2. NISAMs were extracted from water using activated carbon cartridges (Enviro-Clean EPA Method 521 SPE Columns, United Chemical Technologies) and analyzed by liquid chromatography tandem mass spectrometry (LC-MS/MS, Waters AcQuity H-Class Plus Binary System and Waters Xevo TQ-S Micro, United States), following the method described in Li et al. (2023), see more details in Table S4.^18^ The method detection limits (MDLs) and method quantitation limits (MQLs) for the target DBPs are listed in Table S3.

Water temperature, pH, and free/total chlorine measurements were collected using various methods by each utility, with more details in Text SA2. DOC and TDN were quantified using a total organic carbon and total nitrogen analyzer (Shimadzu TOC-L + TN).^19^ Colored dissolved organic matter (CDOM) absorbance (A) was measured with a Horiba Aqualog ultraviolet–visible spectrophotometer, and absorbance spectra were recorded from 190 to 800 nm. Samples where A was less than 2 at 250 nm were further analyzed using a Tidas-E Base Series diode array spectrophotometer (World Precision Instruments).^20^ Data output from the spectrophotometers were converted to the Napierian absorption coefficient, *a* (m^–1^).^21^ Specific UV absorbance at 254 nm (SUVA_254_; L·mg-C^–1^·m^–1^), an indicator of DOM aromaticity defined as the Decadic absorption coefficient at 254 nm (m^–1^) normalized to DOC (mg-C·L^–1^)^22^ was calculated along with the spectral slope over the range 275–295 nm (*S*_275–295_).^23^ Ammonia was analyzed using the Hach TNT830 Kit. Anions, including fluoride, chloride, bromide, nitrite, nitrate, orthophosphate, and sulfate, were analyzed by EPA Method 300.1 Parts A and B. Microbial cell counts were analyzed using a Cytoflex flow cytometer (Beckman Coulter).

### Calculation of Toxicity Index Values

Cytotoxicity and genotoxicity are common toxicity metrics used in DBP studies to evaluate the toxicity of individual and classes of DBPs.^4^ The typical bioassay for cytotoxicity and genotoxicity of DBPs uses Chinese hamster ovary (CHO) cells.^9^ The toxicity of individual DBPs is assumed to be additive when combined in mixtures, as multiple DBPs often co-occur in real water samples.^4,24^ A recent study using CHO assays concluded that the genotoxicity of DBPs may be antagonistic rather than additive.^25^ Despite this, additivity was assumed for both cytotoxicity and genotoxicity calculations in this study to identify toxicity trends as a function of disinfection schemes, time, and other water quality parameters. This enables comparisons of estimated genotoxicity among the different disinfection schemes by using the same metrics for all. The following equations were used to develop cytotoxicity index values (CTI) and genotoxicity index values (GTI):

1 $$CTI=\overset{n}{\underset{i=1}{\sum }}\frac{{[DBP]}_{i}}{LC_{50i}}\times 10^{6}$$

2 $$GTI=\overset{n}{\underset{i=1}{\sum }}\frac{{[DBP]}_{i}}{50\%TDNA_{i}}\times 10^{6}$$

where [DBP]_*i*_ is the molar concentration of each DBP, LC_50_ (M) is the concentration at which there is 50% reduction in the growth of CHO cells compared to the control, 50% TDNA is the concentration at which 50% of DNA migrated away from the nucleus, and 10^6^ is a normalization factor.^4^ Total calculated DBP toxicity for different samples can be compared, where a higher value indicates higher toxicity. LC_50_ values and 50% TDNA values are included in SI Excel Sheets “Data_Cytotoxicity” and “Data_Genotoxicity”. All values are obtained from Wagner and Plewa (2017),^9^ except for LC_50_ values for HKs, which are from Qiu et al. (2024).^26^

### Statistical Methods

Kruskal–Wallis and Dunn’s tests were conducted to compare DBP medians and toxicity value medians across various subgroups to determine significant differences. All significance test results reported are at least of *p*-value < 0.05. Spearman correlation analyses, redundancy discriminant analyses (RDA), and random forest regression (RFR) were all performed to understand relationships between DBPs and water quality parameters, where correlations were significant for *p*-values < 0.05. All statistical analyses were conducted in *R* using DBP concentration data, in which nondetects and values reported as below the method detection limit (<MDL) or below the method quantification limit (<MQL) were imputed as half of MDL (1/2 MDL) to minimize missing data.^27^

## Results and Discussion

### DBP Occurrence

Results of DBP occurrence across the eight utilities are summarized in Figure 1 and the SI Sheet “Data_DBP”. DBP concentrations were compared between chlorinated and chloraminated systems to identify differences in occurrence and speciation and to determine how disinfectant type influences formation patterns. The total DBP concentrations excluding NISAMs in chlorinated systems ranged from 12.1 to 161 ppb, while in chloraminated systems, they ranged from 3.38 to 447 ppb. The range of total NISAMs in chloraminated systems was 1.51 to 25.9 ppt.

**Figure 1:**
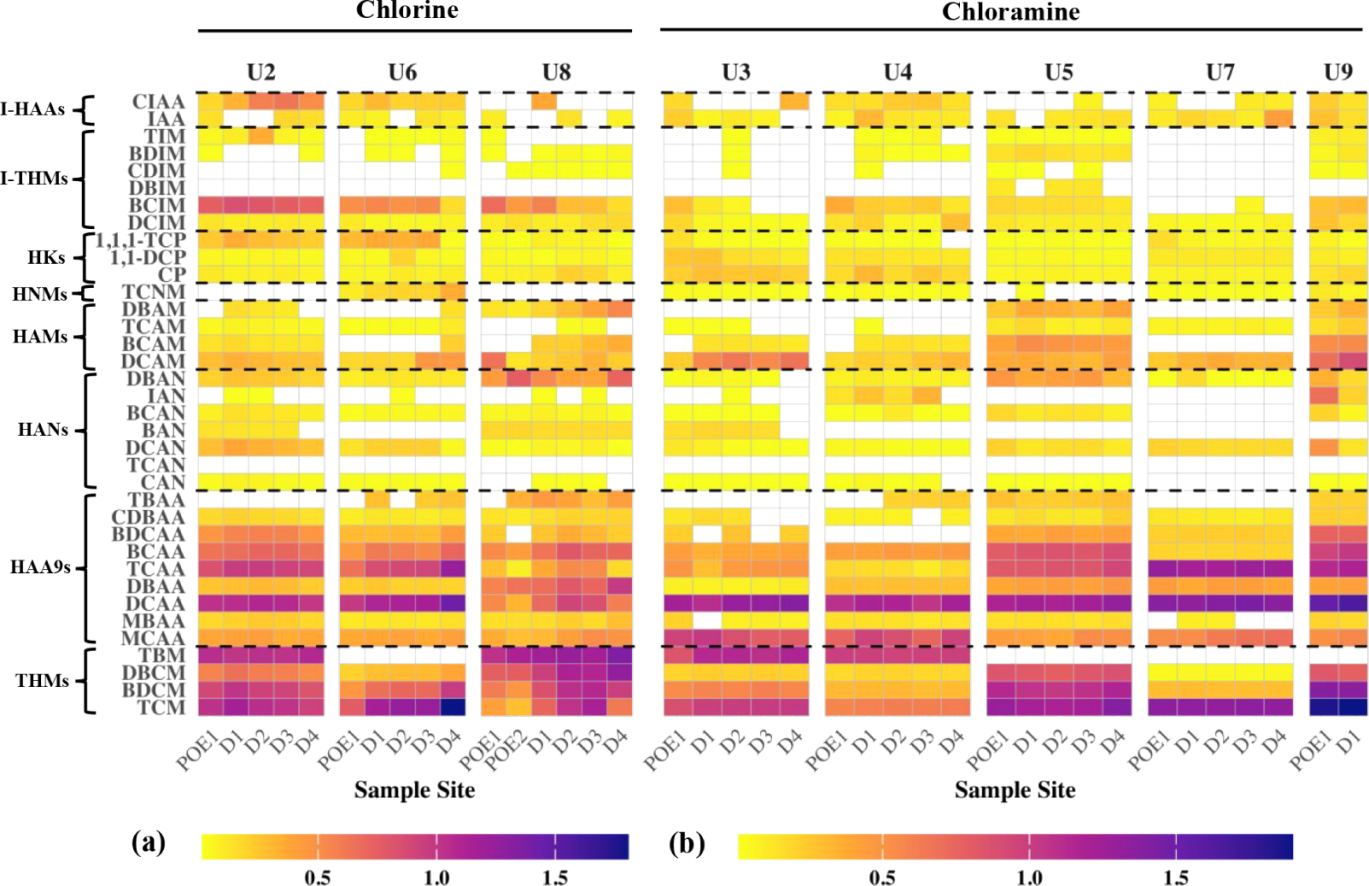
Individual DBP concentrations (ppb in log scale) across all eight utilities: (a) chlorinated systems and (b) chloraminated systems. Values were averaged across seasons. Cells with no color indicate <MQL. Sample sites are ordered by water age. U2, U5, and U7 do not have water age data for each site.

#### THMs and HAAs

The most prevalent DBPs were THMs and HAAs (Figure 1). Of the THMs, trichloromethane (TCM) was the most frequently detected and present at the highest concentrations. Median THMs were significantly higher in chlorinated systems, even though the maximum levels of THMs were observed at U9, a utility using chloramines as the secondary/residual disinfectant, which was related to a high DOC (see later discussion). Goslan et al. (2009) also found elevated THMs in chlorinated systems.^1^ Recent studies of chlorinated DWDS water in China reported median THM levels around 20–30 ppb, which are comparable to the median THMs observed in this study (21 ppb).^14,28^ Of the HAAs, dichloroacetic acid (DCAA) and trichloroacetic acid (TCAA) were the most dominant, followed by bromochloroacetic acid (BCAA) and monochloroacetic acid (MCAA). HAA concentrations were significantly higher in chloraminated systems (30 ppb) compared to chlorinated (20 ppb), which is in contrast to a previous study that found levels in chlorinated systems and chloraminated systems at 44 and 16 ppb, respectively.^1^ Zhou et al. (2019) found lower concentrations of HAAs in chlorinated tap water samples in China (15.3 ppb).^56^ Previous studies have shown that chloraminated systems are more likely to be dominated by dihalogenated HAAs (DXAAs), whereas chlorinated systems contain a mix of mono- (MXAAs), di-, and tri- (TXAAs) HAAs.^1,29^ Consistent with these findings, the present study observed DXAAs as the dominant species in chloraminated systems (DXAAs > MXAAs and TXAAs), while chlorinated systems contained all three classes, with concentrations generally following the order DXAAs > TXAAs > MXAAs.

#### N-DBPs

Dichloroacetonitrile (DCAN) and dibromoacetonitrile (DBAN) were the most frequently detected HANs, at concentrations of up to 3.0 and 10.4 ppb, respectively. In previous studies, DCAN concentrations were higher in chlorinated distribution systems,^14,28^ while DBAN was higher in this current study.^28^ HANs were significantly higher in chlorinated systems, which has been found in other studies.^15,30,31^ The median concentration of six HANs in chlorinated and chloraminated DWDSs in Liew et al. (2016) was 2.8 ppb, which was slightly higher than in this study (1.6 ppb).^15^ Our results showed trichloronitromethane (TCNM) at low concentrations (0.09 ppb), and there were no significant differences between chlorinated and chloraminated systems. Previous studies found similar concentrations of TCNM in distribution system (0.1 ppb).^1,7^ The use of ascorbic acid as a quencher has been shown to degrade TCNM; therefore, the low observed concentrations of TCNM in this study may be partially attributed to this effect. Even so, ascorbic acid was still the most suitable quencher due to its effectiveness at quenching residual disinfectants without interacting with most DBPs or forming additional DBPs.^32^ HAMs occurred at concentrations between 0.15 and 27 ppb in chloraminated systems, which is a wider range than found in other studies; however, the range of concentrations in chlorinated systems (0.057 to 7.1 ppb) was similar to previous studies.^7,15,28,33^

#### HKs and I-DBPs

HKs ranged from 0.02 to 4.1 ppb in chlorinated waters and 0.12 to 2.8 ppb in chloraminated waters, comparable to previously reported values.^28,30,34^ Chloropropanone (CP) and dichloropropanone (1,1-DCP) were more dominant in chloraminated systems, whereas trichloropropanone (1,1,1-TCP) was dominant in chlorinated systems, consistent with other studies that conclude monochloramine cannot sufficiently transform 1,1-DCP to 1,1,1- TCP.^30^ I-THM concentrations (0.08 to 9.7 ppb) were similar or slightly higher than previous research (0.09 to 6.9 ppb).^8^ Bromochloroiodomethane (BCIM) was observed at higher concentrations (1.2 ppb) than dichloroiodomethane (DCIM) (0.2 ppb), contrasting to other studies that identified DCIM at higher concentrations than BCIM.^1,33^ Chlorinated systems had significantly higher I-THM concentrations. Iodinated DBPs are typically lower in chlorinated systems because chlorine fully oxidizes iodide (I^–^) to iodate (IO_3_^–^). In contrast, chloramine is a weaker oxidant, allowing hypoiodous acid (HOI) to persist and form I-DBPs with NOM.^6,11^ Noted that these previous studies were conducted at the laboratory scale using distilled water or iodide-spiked surface waters, where iodide concentrations were controlled. In contrast, this study investigated field occurrence and did not quantify background iodide or iodine-containing organic precursors, which limits the interpretation of the higher I-THMs observed in chlorinated systems. Further, oxidant contact time can also influence the level of I-THM formation,^8^ rendering the comparison of field studies with lab studies challenging. I-HAA median values in chlorinated and chloraminated systems were similar (0.67 and 0.41 ppb, respectively). The I-HAA concentrations in this study were slightly higher than previous studies that identified ranges of 0.002 to 0.09 ppb in chloraminated and 0.033 to 0.67 ppb in chlorinated waters.^8,35,36^

#### NISAMs

Chloraminated utilities were analyzed for nine NISAMs, with NDPhA excluded due to unusually high recoveries that failed the QA/QC criteria (Figure 2). Only data from July to December 2024 are presented here, as limitations in the extraction method affected the first two sampling rounds. As a result, some uncertainty may be associated with the data presented. Most utilities exhibit low NISAM concentrations and few detections. NDMA was the most frequently detected and had an average concentration of around 5.1 ppt, similar to other studies.^37,38^ U4 showed notably elevated NDMA levels, averaging about 13 ppt for sample site D4. NPIP was also elevated at U4 in POE1, and NPYR was elevated at high concentrations at D3 and D4. However, NPIP and NPYR were not detected above the MQL in any other samples.^37,38^ U9 contained only NDBA, which is more commonly detected in drinking water that uses a surface water source rather than groundwater, where rubber materials are a potential precursor.^15^

**Figure 2:**
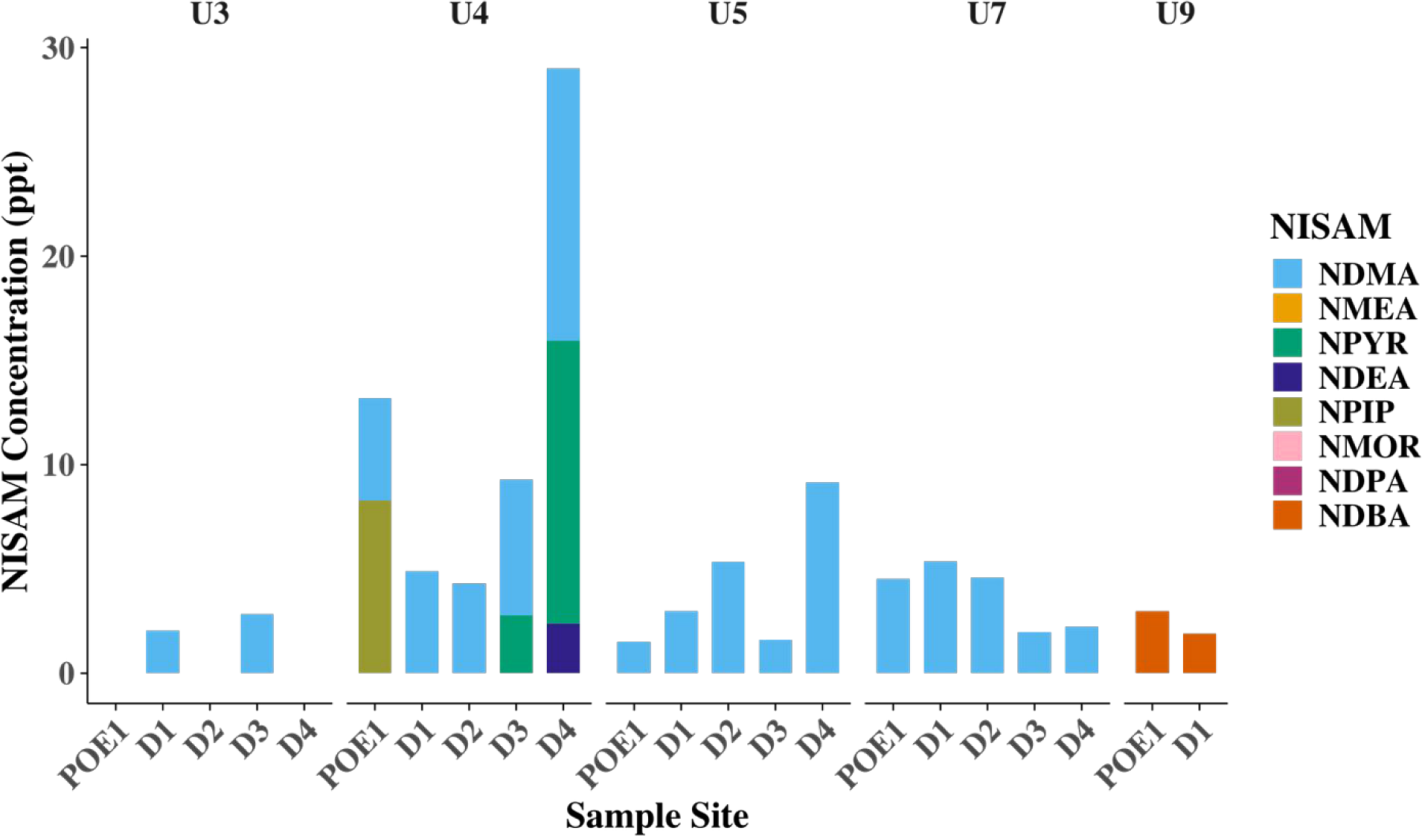
Nitrosamine concentrations for each sampling site averaged over two sampling rounds (July–December 2024). Averages and standard deviation are included in SI Sheet “Data_DBPs”.

### Calculated Toxicity

To further understand the importance of DBPs in DWDSs, cytotoxicity and genotoxicity index values were calculated based on different end points. DBP interactions could be antagonistic or synergistic but in this study, additivity was assumed for both cytotoxicity and genotoxicity for ease of calculations and interpretation.^24,25^ THMs, HKs, and some I-THMs are not genotoxic based on CHO assays; therefore, genotoxic calculations are not computed for those compounds.^8,25^

#### Cytotoxicity

HANs account for the majority of the cytotoxicity; on average across all utilities in this study, HANs contribute to about 56%, HAAs 21%, I-HAAs 16%, and HAMs 7% (Figure 3a and Table S5). Many previous studies identified HANs as the cytotoxicity driver.^4,24,39^ DBAN is the most toxic HAN and dominates cytotoxicity (Figure SA1).^4,39^ MBAA and IAA are the dominant HAAs and I-HAAs, respectively. BCAM and DBAM are the main drivers of HAMs cytotoxicity. These results show that brominated species are of the greatest concern for cytotoxicity even when present at low concentrations. Averaging across all sampling rounds and sample sites, U8, a chlorinated utility, has the highest total cytotoxicity, followed by chloraminated U9 and U5 (Figure 3a). Unregulated DBPs cytotoxicity make up at least 75% of all cytotoxicity, as also observed in previous research.^12,24,40^ To understand the cytotoxicity of regulated DBPs compared to unregulated DBPs, CTI values for the most toxic THM and HAA5 were calculated at the EPA regulation values of 80 and 60 ppb, respectively. TBM at 80 ppb would result in a CTI of 79.8, MBAA, at 60 ppb, results in a CTI of 45000, and the second most toxic HAA5, MCAA, has a CTI value of 784. If MBAA is ignored due to its extremely high CTI value, regulated DBPs overall would have much lower CTI values compared to unregulated DBPs, suggesting that regulated DBPs are not useful proxies for total DBP cytotoxicity.^39,40^

**Figure 3:**
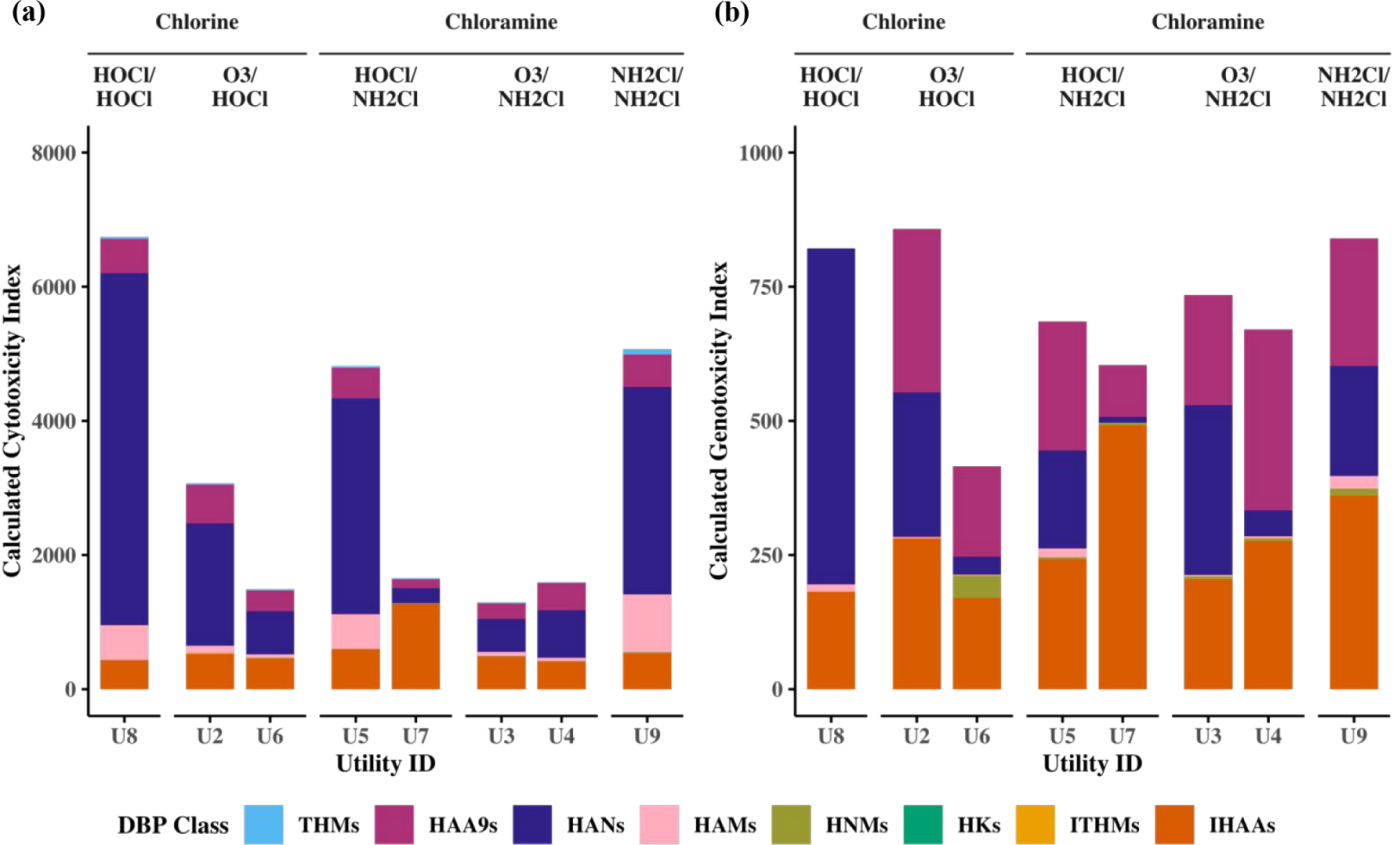
Average calculated (a) cytotoxicity and (b) genotoxicity index values in treated water samples. Values were averaged over sampling rounds and sample locations.

#### Genotoxicity

HAAs are the main driver of genotoxicity on average across all utilities, representing 51% of total genotoxicity, followed by HANs and I-HAAs (26% and 19%, respectively) (Figure 3b and Table S6). Previous studies identified HAAs and HANs as top contributors to genotoxicity.^4,14^ MBAA, MCAA, and DBAA are the main HAAs contributing to genotoxicity. BAN and DBAN are the most dominant HANs, with DBAN being less prevalent in chloraminated systems (Figure SA2). IAA is very dominant across all utilities and is known to be one of the most genotoxic DBPs.^39^ Brominated species are also important drivers of genotoxicity. U8 and U9 have the highest genotoxicity among the utilities (Figure 3b). On average, unregulated DBPs account for 52% of overall genotoxicity across all utilities (Table S6). While previous studies have shown that unregulated DBPs typically contribute more substantially than regulated DBPs, this study found that regulated HAAs contributed appreciably, resulting in a more balanced distribution of genotoxicity between regulated and unregulated DBPs.^25,41^

### Water Quality Parameters

Table 1 summarizes average NOM data for source, POE, and DS samples, and Table S7 provides additional NOM and water quality data. DOC, *a*_254_ (representing aromatic organic matter content in DOC), and SUVA_254_ are all indicators of potential DBP precursor levels.^22,23,42,43^ Spectral slopes (*S*_275–295_, *S*_290–320_, *S*_350–400_, and *S*_350–450_) and slope ratio (*S*_R_ = *S*_275–295_/*S*_350–400_) describe DOM composition such as molecular weight (MW) and source.^23,44–46^ Less negative or shallower *S*_275–295_ (*S*_R_ values less than 1) typically indicate terrestrially derived organic matter and have higher MW. More negative or steeper *S*_275–295_ (*S*_R_ values greater than 1) values suggest microbial/algal-derived, photodegraded organic matter, and have lower MW.^23,44,45^ *S*_275–295_ values are discussed, as they are more suitable than *S*_R_ for characterizing low-absorbance samples such as drinking water.^23^

**Table 1:** Organic Matter Data for Each Source Water, POE, and DS Sample Averaged across Sampling Rounds^a^,^b^

			Source Water Samples				POE Samples			DS Samples		
Utility		Source Water	*DOC*	*a*_254_	*SUVA*	*S*_*275–295*_	*DOC*	*a*_254_	*SUVA*	*DOC*	*a*_254_	*SUVA*
**HOCl**	U2	Surface	1.880	2.924	0.788	–0.028	1.453	2.015	0.631	1.478	1.858	0.605
	U6	Surface	1.465	5.176	1.557	–0.022	1.433	2.062	0.737	1.633	1.89	0.632
	U8	Surface	2.593	11.747	2.172	–0.019	1.130	2.256	1.110	1.051	2.432	1.155
		Ground	0.985	3.042	1.653	–0.020						
**NH**_**2**_ **Cl**	U3	Surface	2.507	20.113	4.037	–0.017	2.470	8.695	1.679	2.449	7.335	1.404
	U4	Blend	5.888	35.909	2.594	–0.023	2.783	7.982	1.249	2.761	8.377	1.314
	U5	Ground	3.615	23.007	1.964	–0.020	2.543	11.465	1.567	3.028	11.628	1.528
	U7	Surface	1.003	8.605	3.979	–0.016	1.995	8.882	3.589	2.129	8.935	3.558
	U9	Ground	7.065	44.207	2.694	–0.023	4.953	24.157	2.116	4.890	23.877	2.139

a Standard deviation values are listed in Table S7.

b Units: DOC = mg-C·L^–1^, *a*_254_ = m^–1^, SUVA_254_ = L·mg-C ^–1^·m^–1^, *S*_275–295_ = m·nm^–1^.

Source water DOC, *a*_254_, and SUVA_254_ were significantly greater in the chloraminated systems in this study, suggesting higher loadings of the DBP precursors. U4, U5, and U9 have the highest DOC and *a*_254_ values. U3 and U7 have the largest SUVA_254_ values, indicating that their source water is composed of more aromatic organic matter (Table 1). Utilities with steeper spectral slopes (*S*_275–295_ values), such as U2, may have more microbial/algal-derived organic matter, whereas utilities with shallower spectral slopes (*S*_275–295_ values) like U3 and U7 may be more influenced by terrestrial organic matter.

In both POE and DS samples, DOC was significantly greater in the chloraminated systems, indicating that even after treatment and after passing through the distribution system, there are still high concentrations of DBP precursors. Because chlorine and chloramine absorb at different wavelengths (259 and 331 nm, respectively), their use as secondary disinfectants is expected to affect *a*_254_ and SUVA_254_, making direct comparisons between systems inappropriate.^47^ Disinfection can also impact spectral slope due to bleaching; therefore, inferences will not be made about spectral slope in treated samples.^23^ At all utilities except for U7, DOC and *a*_254_ appear to decrease between source and POE, indicating efficient removal of bulk and aromatic DOC. The increase in DOC from the source to the POE and within the distribution system at U7 may reflect the presence of organic matter introduced during treatment and contributions from biofilms within the distribution system.^48–50^ The differences between POE and DS samples for DOC, *a*_254_, and SUVA_254_ in all systems were not statistically significant, suggesting that DBP precursors did not vary much throughout the distribution systems.

Water pH ranged from 7.15 to 8.90 in chlorinated POE samples and from 7.14 to 8.95 in chlorinated DS samples. In chloraminated samples, the pH in the POE ranged from 8.67 to 9.66 and in the DS ranged from 8.67 to 9.53. Systems using chloramine had slightly higher pH. Free chlorine in chlorinated samples ranged from 1.19 to 2.25 ppm-Cl_2_ in POE and 0.669 to 1.62 ppm-Cl_2_ in DS samples, indicating a loss of chlorine residual in the distribution system. A similar trend was observed for total chlorine in chloraminated systems: 2.30 to 3.93 ppm-Cl_2_ in POE and 1.85 to 3.47 ppm-Cl_2_ in DS. Bromide was below the detection limit in almost all source water and treated water samples, except for U9 POE and U3 and U9 DS samples. Additional data and discussion are provided in Table S7 and Text SA3.

### Relationships among DBPs and Water Quality Parameters

Analyses used both DBP concentrations and cytotoxicity to identify the key drivers of formation and toxicity. Spearman correlations were performed to identify pairwise associations for chlorinated and chloraminated systems (Figures SB1, SB4) and by utility (Figures SB2, SB3, SB5, SB6), reporting only significant results (*r*_s_ ≥ 0.50, *p* ≤ 0.05). Redundancy discriminant analysis (RDA) assessed multivariate relationships among DBPs, water quality, and microbial cells (Figures 4, SA3, SA4, SA7, SA8) using DOC, *a*_254_, and TDN from source/treated waters, *S*_275–295_ from source waters, and pH, temperature, ammonia, and nitrate from treated waters. Treated TDN and source *a*_254_ were excluded from the chloraminated RDA due to multicollinearity. Random forest regression (RFR) identified the most influential predictors for DBPs (Figures SA5, SA6, SA9 and SA10). Together, these complementary approaches provide a more comprehensive and robust framework for identifying the key water quality drivers of DBP formation and toxicity rather than using one analysis alone.

**Figure 4:**
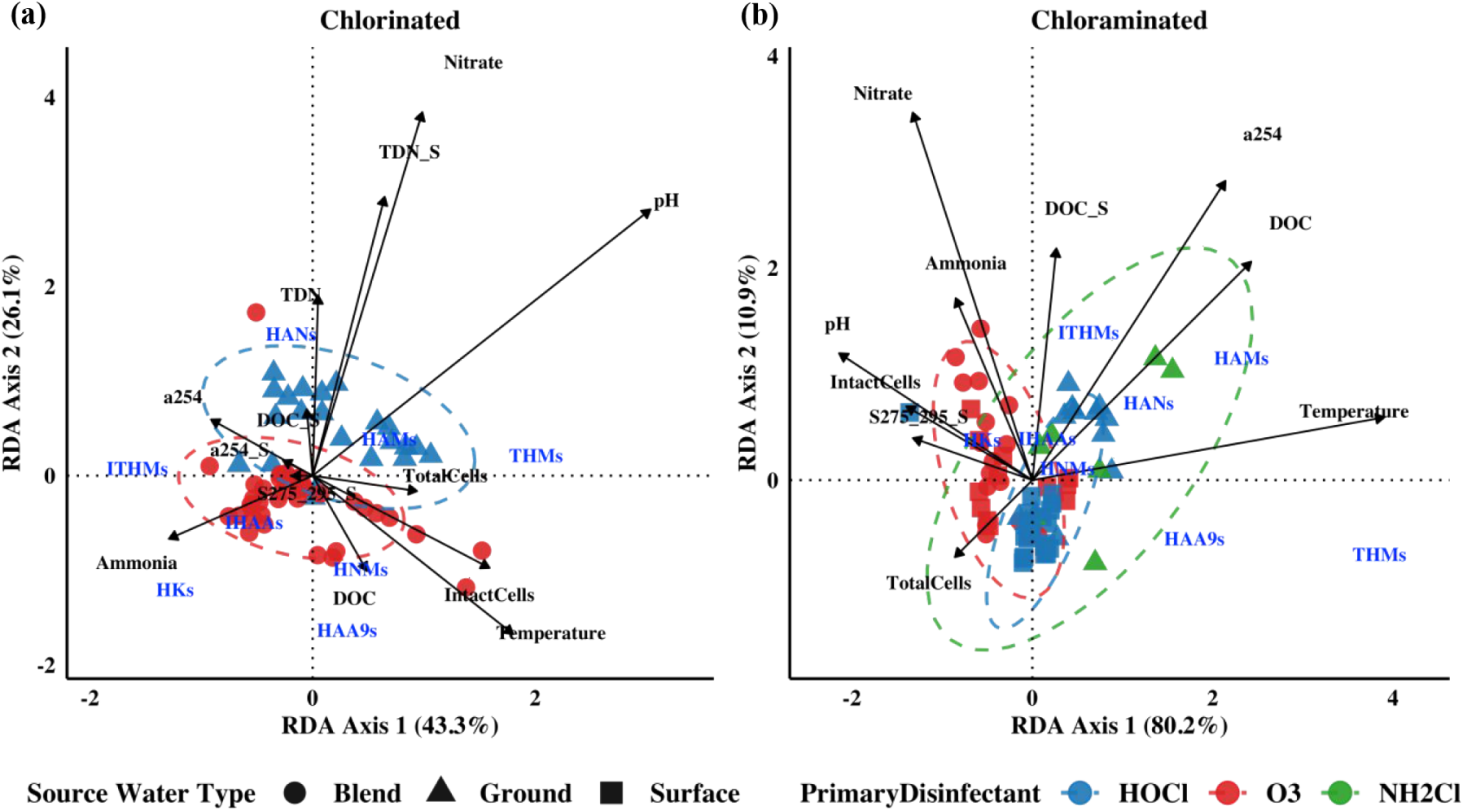
RDA biplot of water quality parameters and DBP concentrations grouped based on classes in (a) chlorinated and (b) chloraminated drinking water samples.

#### DBPs

In chlorinated and chloraminated systems, positive correlations were observed for both concentration and cytotoxicity between THMs and HAMs, THMs and HAAs, and HAMs and HAAs (Figures SB1–SB6). These relationships are consistent with the literature suggesting that HAAs degrade into THMs,^4,7,30^ and HAMs hydrolyze into HAAs.^51^ Additionally, positive correlations between concentrations of HANs and HAMs were observed at some chloraminated utilities, as expected because HANs hydrolyze into HAMs.^15,28,51^ THMs, HAAs, and HAMs showed positive correlations with total DBP concentrations in both chlorinated and chloraminated systems, while HANs correlated with total DBPs only in chloraminated systems. Although these findings suggest that THMs and HAAs could serve as indicators of N-DBP formation and toxicity, HAN cytotoxicity showed the strongest correlation with total cytotoxicity across both system types (Figures SB1b, SA4, SB4b, and SA7).

#### Organic Matter Parameters

In chlorinated systems, DOC (source/treated), source *S*_275–295_, and *a*_254_ were significant predictors at the individual DBP level, highlighting their influence on specific DBPs (Figure SA4), consistent with trends observed in cytotoxic RDAs (Figure SA3b). In the RFR, HANs, HNMs, and I-HAAs were driven by source *S*_275–295_ (Figure SA5e,f and i); however, this trend was not identified in the cytotoxicity RFR (Figure SA6e). Spearman correlation analysis revealed strong positive correlations between DOC and DBP concentrations at U2 (Figure SB2a). Despite these relationships, none of the organic matter parameters emerged as key predictors in the overall RDA, suggesting that the influence of organic matter on DBPs in chlorinated systems is utility and compound specific (Figure 4a).

In chloraminated systems, the RDAs identified DOC (source/treated) as one of the most influential parameters, supported also by the RFR, where DOC was a top predictor for multiple DBPs (Figures 4b, SA9). Specifically, DOC was influential in HANs and HAMs formation, further confirmed by positive correlations at several utilities (Figure SB5a,b,d). Correlations between DOC and N-DBPs, along with cytotoxicity RDAs linking DOC to HAN toxicity, highlight DOC’s role in both DBP formation and toxicity (Figure SA7). In the RDA for individual DBPs, DOC was associated with variation in several HAA species, which may contribute to their elevated levels in chloraminated systems (Figure SA8).

DOC composition and properties may differ between the different source waters; however, concentrations of DOC have been identified as a key factor influencing DBP concentrations at the utility level in chlorinated systems and overall in chloraminated systems. Therefore, DOC-normalized DBP concentrations were calculated using source water DOC. In this study, elevated DOC appeared to be the primary factor associated with higher DBP concentrations in chloraminated systems compared to chlorinated systems. After normalization, differences between disinfectants for HAAs and I-HAAs disappeared, confirming DOC’s role, consistent with RDA and RFR findings (Figure SA11f,n). In contrast, THMs, I-THMs, and HANs remained higher in chlorinated systems (Figure SA11d,h and l). Higher HAN levels in chlorinated systems also resulted in greater cytotoxicity. For HAMs and total DBPs, no differences were observed initially; however, after normalization, HAMs and total DBPs were higher in chlorinated systems, suggesting that differences in DOC may have obscured these trends before normalization (Figure SA11a,b,i,j).

#### Nitrogen Parameters

In chlorinated systems, TDN and nitrate (source/treated) were influential in DBP formation (Figure 4a). TDN and nitrate were negatively correlated with DBPs at U2 and U6 but positively associated with HANs at U8, as shown by their arrows pointing away from O_3_/HOCl samples (U2, U6) and toward HOCl/HOCl samples (U8) in Figure 4a. The RFR also supports TDN and nitrate as strong predictors of N-DBPs (Figure SA5). Cytotoxicity correlations, RDAs, and RFR confirm the role TDN and nitrate play in HAN toxicity (Figures SB3b–c, SA3 and SA6).

In chloraminated systems, both ammonia and nitrate were significant in the RDA, correlation analysis, and RFR (Figure 4b, SB5, SA9). Ammonia, known to promote N-DBP formation,^52,53^ showed positive correlations with HANs and HNMs and was a predictor of HANs in RFR (Figures SB5, SA9e). Nitrate was a strong RFR predictor and negatively correlated with HNMs, despite literature suggesting it may enhance HNM formation (Figures SB5, SA9f).^53^ Nitrate was also a significant factor in N-DBP cytotoxicity (Figure SA7).

#### pH and Temperature

In chlorinated systems, pH emerged as a key driver, particularly for HKs and I-THMs, which were positioned opposite to pH in the RDA, consistent with negative correlations suggesting their degradation into more stable DBPs at higher pH (Figure 4a, SB1a).^52^ Temperature strongly influenced TCAA, BDCAA, and TCM (Figure SA4), but DBP concentrations without and with DOC normalization indicate that the differences are driven by DOC rather than temperature. Since temperature and pH were not influential for HANs, they had little impact on cytotoxicity. In chloraminated systems, pH and temperature were also influential, with THMs and HAAs aligning with temperature (Figures 4b, SA8).^15,28^ However, after DOC normalization, no temperature differences remained, indicating that DOC was the primary driver. RFR identified temperature as a strong predictor of HAN formation and cytotoxicity (Figures SA9e and SA10e), but DOC normalization also revealed that DOC drove these differences, confirming its role in chloraminated system cytotoxicity.

#### Microbial Cells

Relationships between microbial cells and DBPs could suggest that biomass-derived organic matter may serve as a potential DBP precursors. However, across all systems, total and intact cell counts were not influential in class-level RDAs, and in the RFRs, only a few DBPs had total or intact cells as top predictors (Figures SA5, SA9). In contrast, Spearman correlations revealed utility-specific associations between total and intact cells and various DBP classes (Figures SB2, SB5). Taken together, the results indicate that microbial cells were not broadly important drivers of DBP formation across all utilities, but in certain cases, they may contribute as precursors within individual systems.

The correlations identified among DBP classes reflect known degradation pathways and suggest that while THMs and HAAs are correlated with N-DBP concentrations, HANs are the strongest cytotoxicity indicator. DOC was the dominant parameter that influenced DBP formation and cytotoxicity in chloraminated systems, while organic matter was less influential in chlorinated systems. TDN and nitrate were key drivers of N-DBP formation and cytotoxicity in chlorinated systems, with nitrate and ammonia playing important roles in chloraminated systems. Temperature appeared to be influential; however, after DOC normalization, it was revealed that DOC was the primary driver. Microbial cells had little influence overall but appeared to be more relevant at the utility level. Using Spearman correlations, RDAs, and RFR together provides a comprehensive assessment of water quality parameter impacts on DBP formation and cytotoxicity.

### Engineered Factors Affecting DBP Formation

This section explores two engineered factors, the disinfectant scheme and distribution system location, to determine their influence on DBP formation and toxicity. As discovered in the previous section, DOC was a strong factor influencing DBP concentrations, particularly for chloraminated systems. For that reason, DOC-normalized DBP concentrations were used to determine differences between primary disinfectants.

#### Disinfectant Scheme

There were five unique disinfectant schemes represented among the eight utilities in this study, using combinations of chlorine (HOCl), ozone (O_3_) or monochloramine (NH_2_Cl) as the primary disinfectant and HOCl or NH_2_Cl as the secondary disinfectant. As discussed in the previous section, after accounting for DOC, total DBPs, THMs, HANs, and I-THMs were higher in chlorinated systems, and HAMs were higher in chloraminated systems (Figure SA11b,d,h,l). Due to increased HANs in chlorinated systems, cytotoxicity was also elevated; however, there were no differences for genotoxicity.

In chlorinated systems, after DOC normalization of concentrations, O_3_ as the primary disinfectant increased HAAs, HNMs, HKs, and I-THMs (Figure 5b,e–g), consistent with past studies reporting higher HKs and TCNM with O_3_/HOCl.^15,30^ HOCl/HOCl formed more HANs and thus had significantly higher cytotoxicity and genotoxicity than did O_3_/HOCl, consistent with the clustering of N-DBPs with HOCl samples in the RDAs (Figures 4a, 5c, SA3, SA4).

**Figure 5:**
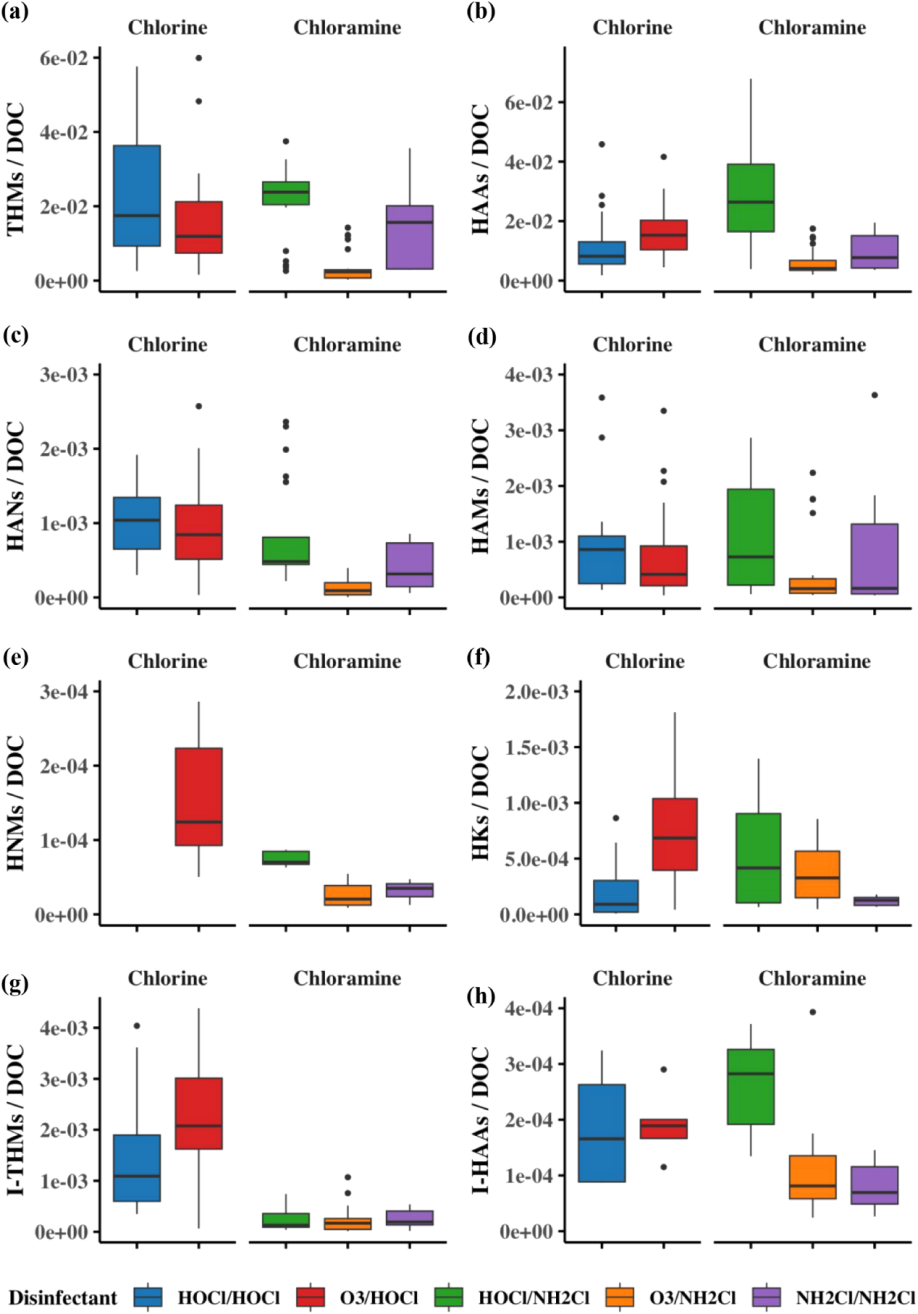
Comparison between DOC-normalized concentrations for DBP classes by different disinfectant schemes: (a) THMs, (b) HAAs, (c) HANs, (d) HAMs, (e) HNMs, (f) HKs, (g) I-THMs, and (h) I-HAAs. Some outliers removed for visualization.

In chloraminated systems, after DOC normalization, THMs were greater when HOCl or NH_2_Cl were used as primary oxidants compared to O_3_ (Figure 5a). Concentrations of HAAs were higher with HOCl compared with both O_3_ and NH_2_Cl (Figure 5b). HANs, HNMs, and HAMs were elevated when HOCl was used compared to that with O_3_ (Figure 5c–e). This finding indicates that HOCl as a primary oxidant can drive both cytotoxicity and genotoxicity, as identified for chlorinated systems too. No differences were observed for HKs, I-THMs, I-HAAs, and NISAMs. Differences in NISAMs, especially at U4, might be driven by the use of polyDADMAC for zebra mussel control (Figure 2).^38,54^ In chloraminated systems, total cytotoxicity was greater when either HOCl or NH_2_Cl was used as a primary disinfectant compared to that with O_3_. Utilities using O_3_/NH_2_Cl had the lowest cytotoxicity, consistent with previous findings that this combination reduces cytotoxicity (Figure 3).^39^ No differences were observed in genotoxicity between primary disinfectants.

Overall, in chlorinated systems, the primary disinfectant, O_3_ (as the primary disinfectant), produced more HAAs, HNMs, HKs, and I-THMs than HOCl, while HOCl increased HANs, which led to higher overall toxicity. In chloraminated systems, HOCl/NH_2_Cl increased THMs, HAAs, HANs, HAMs, and HNMs compared with other disinfectant schemes. Overall cytotoxicity was higher in NH_2_Cl/NH_2_Cl, and HOCl/NH_2_Cl compared to that in O_3_/NH_2_Cl, largely driven by HANs.

#### Distribution System Location

DBP formation can be influenced by the water age, reflecting the dynamic nature of distribution systems and the importance of assessing DBPs under real-world conditions. Water age for U2, U5, and U7 was not available, and U9 only had two sample site locations; therefore, these utilities will not be discussed in this section.

Chlorinated systems, U6 and U8, have water ages ranging from 0 to 75 h and 0 to 168 h, respectively. At both utilities, THMs and HAAs slightly increased with water age (Figure SA12), aligning with previous findings for THMs but disagreeing with studies that noted HAA degradation over time.^1,13,14^ These increases were particularly noted for TCM, DCAA, and TCAA at U6 (Figure 1). HANs were stable at U6 but fluctuated at U8. Cytotoxicity and genotoxicity trends at U8 followed closely the HAN concentrations over time (Figure SA13), while at U6, both cytotoxicity and genotoxicity were stable over time. TDN and nitrate at U8 followed a very similar pattern to HANs as well (Figure SA14g,h). HAMs gradually increased over time at both utilities. TCNM increased at U6 but was <MQL for all sample sites at U8 (Figure SA12). The trend in pH at U6 mimicked the increasing trend in TCNM (Figure SA14b). HKs declined at U6 but remained stable at U8 (Figure SA12).^14^ I-THMs consistently decreased, in contrast to a previous study that observed increases along the distribution system, likely due to elevated iodide concentrations in the source water.^55^ The lack of iodide measurements in this study limits the interpretation of why I-THMs decreased in the distribution systems. I-HAAs were variable but remained low overall.

In chloraminated systems, U3 and U4, water age ranges from 0 to 96 h and 0 to 68 h, respectively. THM levels remained stable across water age, consistent with studies reporting no increase in THMs in such systems (Figure SA12).^1^ HAAs had slight increases. HANs were constant over time, differing from studies that reported increases.^34^ Constant HANs over time explain the consistency of overall toxicity as a function of water age (Figure SA13). HAMs increased at U3 but remained constant over time at U4. TCNM remained low over time. HKs and I-THMs declined over time (Figure SA12).^14^ I-HAAs were variable but at low concentrations, similar to chlorinated systems. NDMA increased with water age at U4, which supports findings of higher NDMA levels further from treatment plants.^38,54^ Water quality parameters in chloraminated utilities did not vary enough over time to explain any differences in DBPs (Figure SA14).

In both chlorinated and chloraminated systems, THMs, HAAs, HANs, HNMs, and HAMs remained largely stable across the water age, with a few utility-specific increases. I-THMs, HKs, and I-HAAs showed greater variability but remained at low concentrations. Toxicity patterns over water age were utility-specific but driven by the main toxicity driver identified in this study, HANs. Because DBPs showed little change with water age, it was difficult to use variations in water quality parameters over time to explain DBP trends.

## Environmental Implications

This study examined key factors influencing DBP formation across eight drinking water distribution systems in the United States. To capture the complexity of DBP formation, three statistical analytical approaches were applied and compared: Spearman correlations, redundancy analysis, and random forest regression. As a result, DOC emerged as a major driver in chloraminated systems, where higher DOC levels were associated with increased DBP concentrations and toxicity. In chlorinated systems, the influence of individual water quality parameters was less consistent, varying by compound class and utility, although the disinfectant scheme was identified as a key driver. In chloraminated systems, disinfectant effects were compound class-specific. Water age showed a limited impact on DBP formation or toxicity. These findings highlight the value of combining multiple statistical techniques and underscore the need for tailored monitoring and predictive strategies, particularly as machine learning models gain traction for reducing experimental burdens in DBP research.

Despite these insights, this study has a few limitations. Incomplete NISAM data due to issues with extraction and analytical methods during the early stage of the study constrained our ability to assess their relationships with water quality parameters and associated toxicity. The absence of organic nitrogen data limited a deeper investigation into nitrogenous DBP formation. Additionally, a more detailed characterization of individual source waters would have helped clarify the drivers of specific DBPs, especially in systems where brominated DBPs were present despite nondetectable bromide levels. The lack of iodide measurements similarly restricted the interpretation of I-THM formation, particularly in chlorinated systems. To protect the anonymity of each utility, a detailed analysis of the impact of different treatment processes on DBP formation was not conducted, which reduced the capacity to fully understand the engineered factors driving the variability in DBP formation across systems. Furthermore, missing water age data for some utilities limited the temporal analyses. Nevertheless, the data set compiled in this study is robust and offers potential for future applications, particularly for developing predictive models using machine learning to support data-driven DBP management and regulatory strategies.

## Supplementary Material







## Notes

Sampling, experimental methods, and water quality parameters (Text SA1–SA3). Additional figures (Figures SA1–SA14) (PDF)
Additional figures for Spearman Correlations (Figures SB1–SB6) (PDF)
Additional tables for method details and data (Tables S1–S7) (XLSX)
