# Supplementary material for: Identification of Factors Influencing Variability in Disinfection Byproducts and Their Toxicity in Chlorinated and Chloraminated Drinking Water Distribution Systems across the United States
Source: Environ Sci Technol. 2025 Dec 31;60(1):1241–52. doi: 10.1021/acs.est.5c12121 (PMC12810234; doi:10.1021/acs.est.5c12121)
Supplement: Supplementary file 1 [file es5c12121_si_001.pdf]

Supporting Information A

Identification of factors influencing variability in disinfection byproducts and their toxicity in chlorinated and chloraminated drinking water distribution systems across the United States

Samantha DiLoreto[1]; Huanqi He[1,2]; Jinhao Yang[1]; Patrick Milne[3]; Jiaqi Li[1]; Christopher A. Impellitteri[4]; Aron Stubbins[3,5,6]; Ameet Pinto[1,7]; Ching-Hua Huang[1,*]

[1] School of Civil and Environmental Engineering, Georgia Institute of Technology, Atlanta, Georgia, 30332, USA

[2] School of Science and Engineering, Benedict College, Columbia, South Carolina, 29204, USA

[3] Department of Chemistry and Chemical Biology, Northeastern University, Boston, Massachusetts, 02115, USA

[4] The Water Tower, Buford, Georgia, 30519, USA

[5] Department of Marine and Environmental Sciences, Northeastern University, Boston, Massachusetts, 02115, USA

[6] Department of Civil and Environmental Engineering, Northeastern University, Boston, Massachusetts, 02115, USA

[7] School of Earth and Atmospheric Sciences, Georgia Institute of Technology, Atlanta, Georgia, 30332, USA

* Corresponding author: Ching-Hua Huang (ching-hua.huang@ce.gatech.edu)

Table of Contents

Text SA1. Sample Location and Collection.

Text SA2. Quantification of DBPs and Water Quality Parameters.

Text SA3. Additional Water Quality Parameters.

Figure SA1. Individual DBP cytotoxicity index values (CTI) across all eight utilities: (a) chlorinated systems and (b) chloraminated systems. Values were averaged across seasons and sample sites are ordered by water age. Cells with no color indicate no toxicity value was calculated. U2, U5, and U7 do not have water age data for each site.

Figure SA2. Individual DBP genotoxicity index values (GTI) across all eight utilities: (a) chlorinated systems and (b) chloraminated systems. Values were averaged across seasons and sample sites are ordered by water age. Cells with no color indicate no toxicity value was calculated. U2, U5, and U7 do not have water age data for each site.

Figure SA3. RDA biplot of water quality parameters and DBP cytotoxicity in chlorinated systems grouped based on (a) classes of DBPs and (b) individual DBPs.

Figure SA4. RDA biplot of water quality parameters and individual DBP concentration in chlorinated systems.

Figure SA5. Random Forest variable relative importance graphs for chlorinated systems for the following DBP classes: (a) Total DBPs, (b) THMs, (c) I-THMs, (d) HKs, (e) HANs, (f) HNMs, (g) HAMs, (h) HAA9s, and (i) I-HAAs.

Figure SA6. Random Forest variable relative importance graphs for chlorinated systems for the cytotoxicity of the following DBP classes: (a) Total DBPs, (b) THMs, (c) I-THMs, (d) HKs, (e) HANs, (f) HNMs, (g) HAMs, (h) HAA9s, and (i) I-HAAs.

Figure SA7. RDA biplot of water quality parameters and DBP cytotoxicity in chloraminated systems grouped based on (a) classes of DBPs and (b) individual DBPs.

Figure SA8. RDA biplot of water quality parameters and individual DBP concentration in chloraminated systems.

Figure SA9. Random Forest variable relative importance graphs for chloraminated systems for the following DBP classes: (a) Total DBPs, (b) THMs, (c) I-THMs, (d) HKs, (e) HANs, (f) HNMs, (g) HAMs, (h) HAA9s, and (i) I-HAAs.

Figure SA10. Random Forest variable relative importance graphs for chloraminated systems for the cytotoxicity of the following DBP classes: (a) Total DBPs, (b) THMs, (c) I-THMs, (d) HKs, (e) HANs, (f) HNMs, (g) HAMs, (h) HAA9s, and (i) I-HAAs.

Figure SA11. Comparison between DBP concentrations and DOC-normalized concentrations for DBP classes by secondary disinfectant. (a) and (b) Total DBPs, (c) and (d) THMs, (e) and (f) HAAs, (g) and (h) HANs, (i) and (j) HAMs, (k) and (l) I-THMs, and (m) and (n) I-HAAs. Some outliers were removed for visualization. Classes with low concentrations were not included in this figure.

Figure SA12. Average DBP concentration across water age* (sample site used as proxy). * = Only utilities with provided water age data were included.

Figure SA13. Average total DBP (a) cytotoxicity and (b) genotoxicity across water age* (sample site used as proxy). * = Only utilities with provided water age data were included.

Figure SA14. (a) Residual oxidant (b) pH (c) temperature, (d) DOC, (e) UV_{254}, (f) slope ratio, (g) TDN, (h) nitrate, and (i) ammonia across sample site (proxy for water age*). Values averaged across sampling rounds. * = Only utilities with provided water age data were included.

Text SA1. Sample Location and Collection. Samples in this study were collected from eight drinking water treatment utilities (named U2-U9) across the United States, representing a broad range of common drinking water and treatment characteristics. Table S2 includes relevant information regarding each utility's size, source water type, and disinfection schemes. The eight utilities span a wide size range from 3 MGD to 420 MGD. Source water types include surface water, groundwater, and a blend of surface and groundwater. U2, U6, and U8 are considered chlorinated systems in this study as they use chlorine (HOCl) as their secondary disinfectant. U2 and U6 use ozone (O_3) as their primary disinfectant and U8 uses HOCl. U3, U4, U5, U7, and U9 are considered chloraminated systems in this study as they use chloramine (NH_2Cl) as their secondary disinfectant. U3 and U5 use O_3 as their primary disinfectant, U5 and U7 uses HOCl, and U9 uses NH_2Cl.

Drinking water samples were collected from January 2024 to December 2024. Personnel at each utility collected water samples using instructions and bottles provided by the research team from the source water, treated water from the point of entry (POE) to the distribution system, and several locations within the distribution system (DS). The number of samples and water age of the DS samples varied among the utilities. All utilities except for U8 and U9 provided four DS samples and one POE sample. U8 provided four DS samples and two POE samples. U9 provided one DS and one POE sample. Samples were collected during four rounds throughout the year: round 1 (January and February), round 2 (April and May), round 3 (July and August), and round 4 (October and November). At each sampling location, two 125-mL amber glass bottles were filled, ensuring they were headspace free. One bottle contained ascorbic acid (0.0043 g) for analysis of HAAs and I-HAAs (EPA Method 552.3). The other bottle contained ascorbic acid (0.0043 g) and phosphate buffer (to adjust pH to 4.5-5.5) for analysis of THMs, I-

THMs, HKs, HANs, TCNM, and HAMs (EPA Method 551.1). Personnel at chloraminated utilities filled a 1-L amber glass bottle containing ascorbic acid (0.0329 g) for nitrosamine analysis. Additional field duplicates and laboratory field sample matrix samples were collected during each round of sampling for each utility. Samples were shipped on ice (4°C) to Georgia Institute of Technology (Atlanta, Georgia, USA) for DBP analysis.

During each round of sample collection, utility workers collected temperature, pH, and either free or total chlorine measurements for each sample location. Samples were also collected for analysis of dissolved organic carbon (DOC), absorbance at UV 254 nm, ammonia, total dissolved nitrogen (TDN), and various other organic matter parameters and shipped to Northeastern University (Boston, Massachusetts, USA) for analysis. Anion analysis samples were collected and shipped to The Water Tower (Buford, Georgia, USA).

Text SA2. Quantification of DBPs and Water Quality Parameters.

THMs, I-THMs, HKs, HANs, TCNM, and HAMs. THMs, I-THMs, HKs, HANs, TCNM, and HAMs were extracted by liquid-liquid extraction (LLE) with MTBE following EPA Method 551 with minor modifications. To briefly explain, 50 mL of sample was added to a 60-mL amber glass vial, decafluorobiphenyl was added as a surrogate standard (10 ppb). Three ml of MTBE were added to each sample. Immediately following the addition of 10 g of sodium sulfate, samples were shaken for four minutes. The MTBE portion of the sample was pipetted into a GC vial. Procedural calibration standards, laboratory reagent blanks (LRB), field reagent blanks (FRB), field duplicates (FD), and laboratory fortified sample matrices (LFSM) were extracted along with samples for QAQC. Following extraction, samples were analyzed using gas-chromatography coupled with electron capture detection (GC-ECD, 7890A GC System, Agilent

Technologies, United States) with a J&W DB-1 GC Column (30 m x 0.25 mm x 0.25 μm). The column was held at 35°C for 15 min, heated to 100°C at 10°C /min and held for 2 minutes and then finally increased to 150°C at 5°C /min and held for 3 minutes. Injector and ECD temperatures were 230°C and 260°C, respectively. High purity nitrogen gas was used as the carrier and make-up gas. Calibration curves had at least R^2 of 0.98. Relative percent difference for FDs was less than 25% and percent recovery for LFSMs was between 75% and 125% for averages of 75% and 84% of compounds, respectively. Surrogate recovery was between 75% and 125% for an average of 75% of samples. TBM concentrations were blank subtracted due to overlap with impurity peak in all samples and QAQC samples.

HAAs and I-HAAs. HAAs and I-HAAs were extracted using LLE and acid derivatization following EPA Method 552 with minor modifications. In short, 40 mL of sample was added to a 60-mL amber glass vial, 2-bromobutanoic acid was added as a surrogate standard (10 ppb). 18 g of sodium sulfate was added, and samples were shaken for four minutes. Four mL of MTBE with the internal standard (1,2-dibromopropane, 100 ppb) were added and samples were shaken for three minutes. The top layer of each sample (about 3 mL) was pipetted into a 15 mL glass centrifuge tube. Three mL of 10% sulfuric acid in methanol was added to each sample and then heated at 50°C ± 2°C for 2 hours ± 10 minutes. After samples cooled to room temperature, 7 mL of a 150 g/L sodium sulfate solution were added and then samples were vortexed. The bottom aqueous layer was removed and disposed of. One mL of a saturated sodium bicarbonate solution was added, and samples were vortexed again. The top layer was pipetted into GC vials. Samples were analyzed using the same GC-ECD and column as mentioned above. The column was held at 40°C for 5 min, heated to 52°C at 5°C/min and held for 5 minutes, then heated to 80°C at

2.5°C/min and held for 2 minutes, next to 150°C at 10°C/min and held for 2 minutes, and finally increased to 185°C at 25°C/min. Injector and ECD temperatures were 210°C and 280°C, respectively. High-purity nitrogen gas was used as the carrier and make-up gas. The same QAQC procedures described above were used for this method as well. Relative percent difference for FDs was less than 25% and percent recovery for LFSMs was between 75% and 125% for averages of 87% and 94% of compounds, respectively. Surrogate recovery was between 75% and 125% for an average of 85% of samples. See Table S3 for the method detection limits (MDLs) and method quantitation limits (MQLs) for all methods.

NISAMs. One L samples were analyzed for NISAM analysis following the method described in Li et al. (2023).[1] Briefly, samples were spiked with deuterated internal standards (NDMA-d6, NDEA-d10, NDPA-d14, NDPhA-d6, 20 ppb in 0.3 mL). Activated carbon cartridges (Enviro-Clean EPA Method 521 SPE Columns, United Chemical Technologies) were precondition with 6 mL dichloromethane, 6 mL methanol, and 15 mL Milli-Q. Next, samples were passed through the cartridges under vacuum at a flow rate of 3-4 mL/min. After drying for 15 minutes under vacuum, the cartridges were eluted with 15 mL dichloromethane (DCM) at a flow rate of 2-3 mL/min. The extracts were collected in 15 mL glass centrifuge tubes and evaporated using nitrogen gas to remove DCM leaving only water. Samples were reconstituted to 0.3 mL using LC-MS grade water. Laboratory reagent blanks (LRB), field reagent blanks (FRB), field duplicates (FD), and laboratory fortified sample matrices (LFSM) were extracted along with samples for QAQC. Calibration standards were prepared following extraction and prior to analysis. Samples were analyzed for nine NISAMs using liquid chromatography tandem mass spectrometry (LC-MS/MS, Waters AcQuity H-Class Plus Binary System and Waters Xevo TQ-S

Micro) in positive electrospray mode with a corona pin (ESCi+). The MDLs for all nine NISAMs ranged from around 0.33 ppt to 1.66 ppt and the MDL for NDMA was 0.42 ppt (see Table S3 for all MDLs and MQLs). Table S4 lists further details about the LC parameters and optimized MS conditions. Average recoveries of the internal standards in samples and average concentrations in LRBs (subjected to SPE) are listed in Table S4. All samples were blank subtracted.

Water Quality Parameters. Free and total chlorine residuals were measured in the field using colorimetric methods consistent with Standard Methods 4500-Cl G (DPD method). The most common approaches included the use of portable Hach colorimeters. Several utilities also used handheld photometers (e.g., HF Scientific Pocket Photometer) following the same standard method. To quantify DOC and TDN, samples were acidified to pH 2 and analyzed using a total organic carbon and total nitrogen analyzer (Shimadzu TOC-L+TN).[2] To determine colored dissolved organic matter (CDOM) absorbance (A), sample (non-acidified) was placed in a 1 cm quartz cell situated in the light path of an Horiba Aqualog ultraviolet-visible spectrophotometer and CDOM absorbance spectra were recorded from 190 to 800 nm. For samples where absorbance (A) measured using a 1 cm cell was less than 2 at 250 nm, samples were further analyzed using a Tidas-E Base Series diode array spectrophotometer (World Precision Instruments) with a 10 cm flow cell. In both cases, ultrapure (MilliQ) water provided a blank. Blank corrected absorbance spectra were corrected for offsets due to scattering and instrument drift by subtraction of the average absorbance between 700 and 800 nm.[3] Data output from the spectrophotometers were in the form of dimensionless absorbance (A) and were subsequently converted to the Napierian absorption coefficient, a (m^{-1}).[4] Specific UV absorbance at 254 nm

($SUVA_{254}$; L mg-C $^{-1}$ m^{-1}), an indicator of DOM aromaticity defined as the Decadic absorption coefficient at 254 nm (m^{-1}) normalized to DOC (mg-C L^{-1})[5] was calculated along with spectral slope over the range 275–295 nm ($S_{275-295}$).[6] To determine microbial cell counts, samples were processed using Cytoflex Flow Cytometer (Beckman Coulter). Samples were stained with Invitrogen™ SYBR Green I (SG) combined with Molecular Probes™ propidium iodide (PI) and then incubated in the dark for at least 15 minutes. Flow cytometric measurements were performed using a 50mW solid-state laser operating at 488 nm wavelength. Green and red fluorescence were detected at 525 ± 40 nm and 690 ± 50 nm, respectively, together with forward (FSC) and side scatter (SSC) signals. Data were analyzed using FlowJo software (FlowJo LLC), where electronic gating was applied to distinguish cell-associated fluorescence from background noise.

Text SA3. Additional Water Quality Parameters.

In chlorinated systems, TDN in POE samples ranged from 0.358 to 1.293 ppm-N and in DS from 0.350 to 0.937 ppm-N. In chloraminated systems, POE samples contained TDN from 0.438 to 1.110 ppm-N and in DS from 0.451 to 1.096 ppm-N. Ammonia in chlorinated systems was very low, on average, 0.020 ppm-N in POE and 0.017 ppm-N in DS samples. In chloraminated systems, ammonia was much higher, 0.587 ppm-N average in POE samples and 0.561 ppm-N in DS samples. Nitrate was higher in chlorinated systems, average 2.510 ppm-N in POE samples and 2.270 in DS samples. In chloraminated systems, the average nitrate concentration in POE samples was 1.238 ppm-N and 1.176 ppm-N in DS samples. Additional values are included in Table S7.

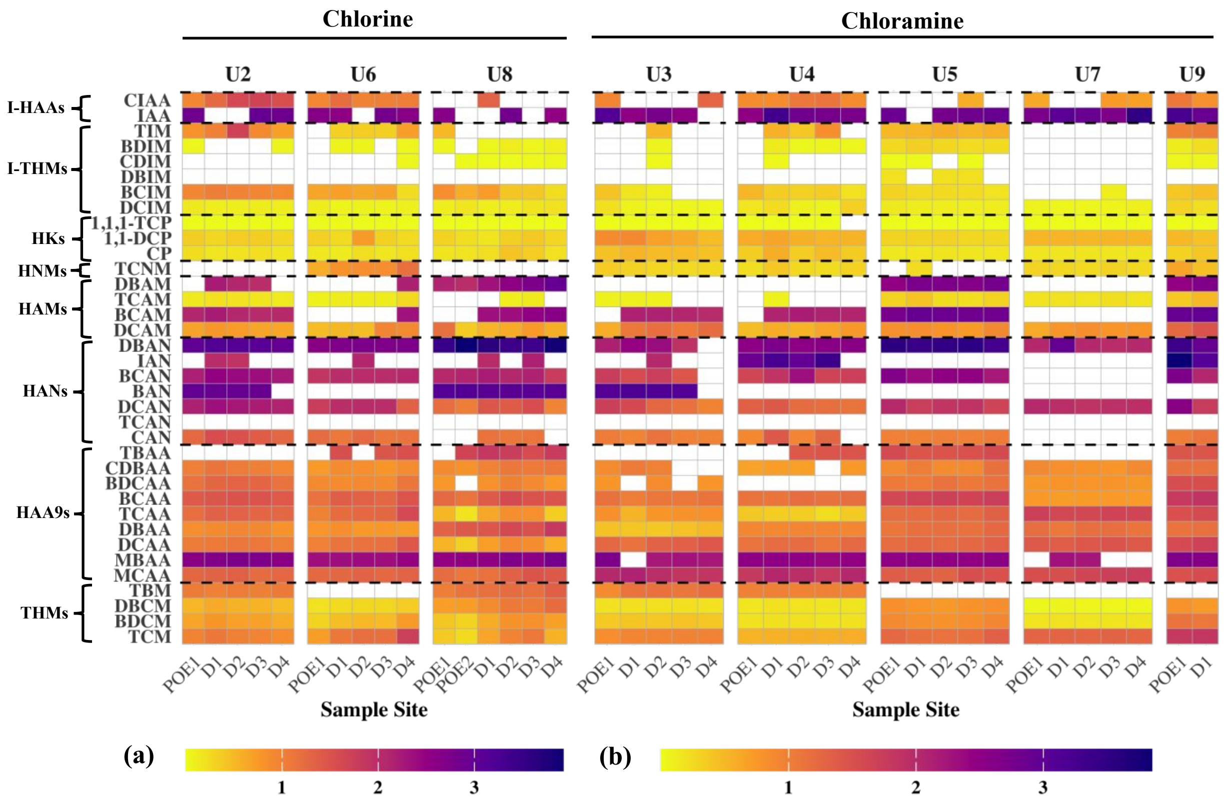

Figure SA1. Individual DBP cytotoxicity index values (CTI) across all eight utilities: (a) chlorinated systems and (b) chloraminated systems. Values were averaged across seasons and sample sites are ordered by water age. Cells with no color indicate no toxicity value was calculated. U2, U5, and U7 do not have water age data for each site.

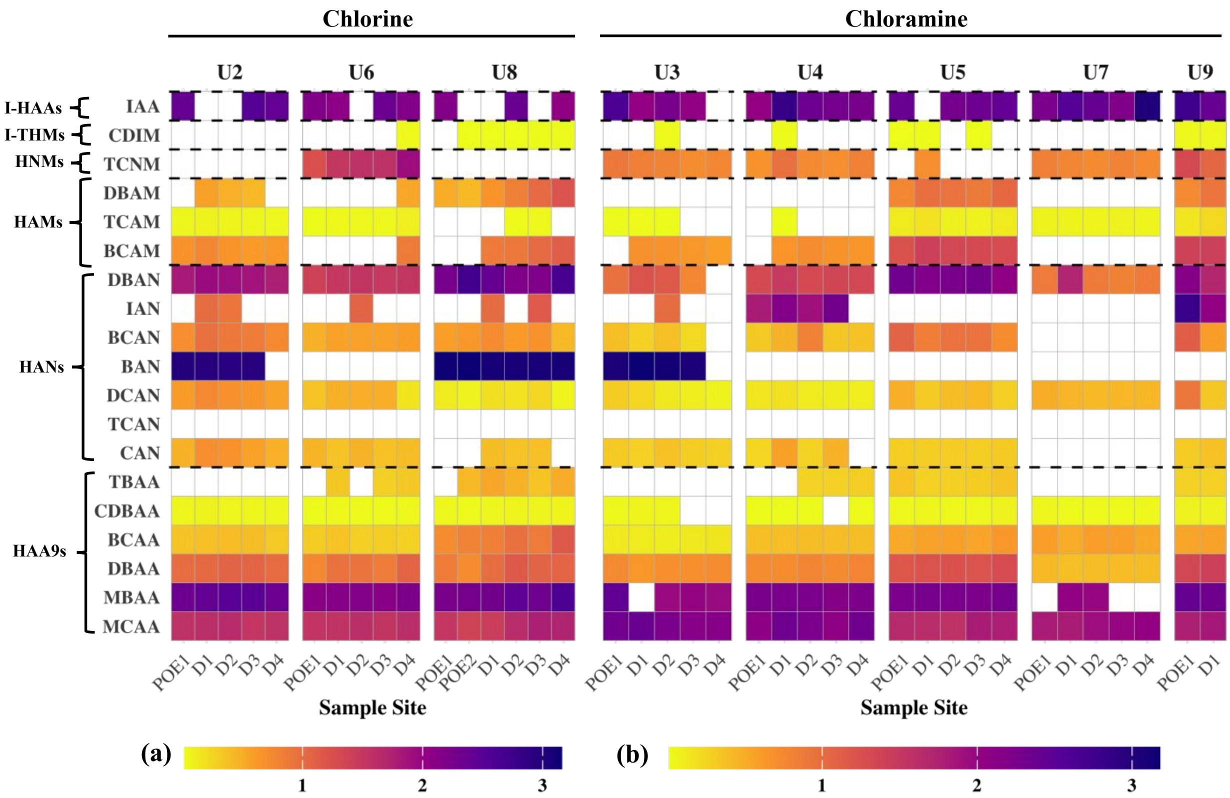

Figure SA2. Individual DBP genotoxicity index values (GTI) across all eight utilities: (a) chlorinated systems and (b) chloraminated systems. Values were averaged across seasons and sample sites are ordered by water age. Cells with no color indicate no toxicity value was calculated. U2, U5, and U7 do not have water age data for each site.

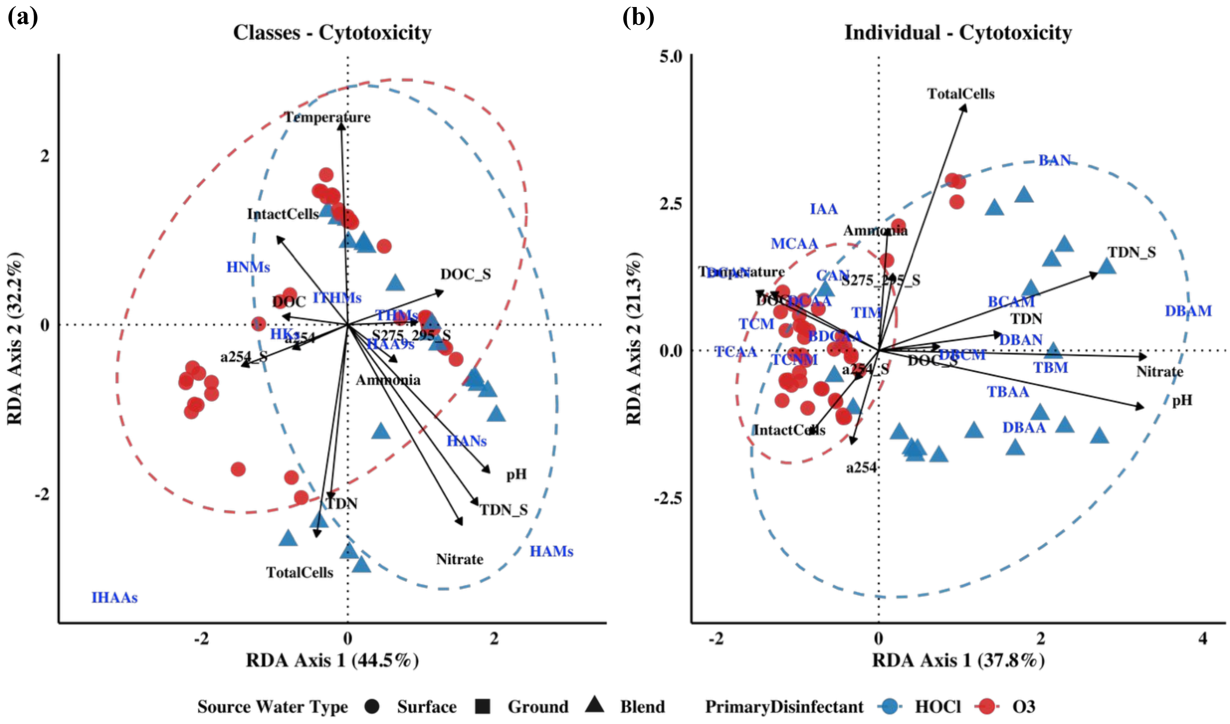

Figure SA3. RDA biplot of water quality parameters and DBP cytotoxicity in chlorinated systems grouped based on (a) classes of DBPs and (b) individual DBPs.

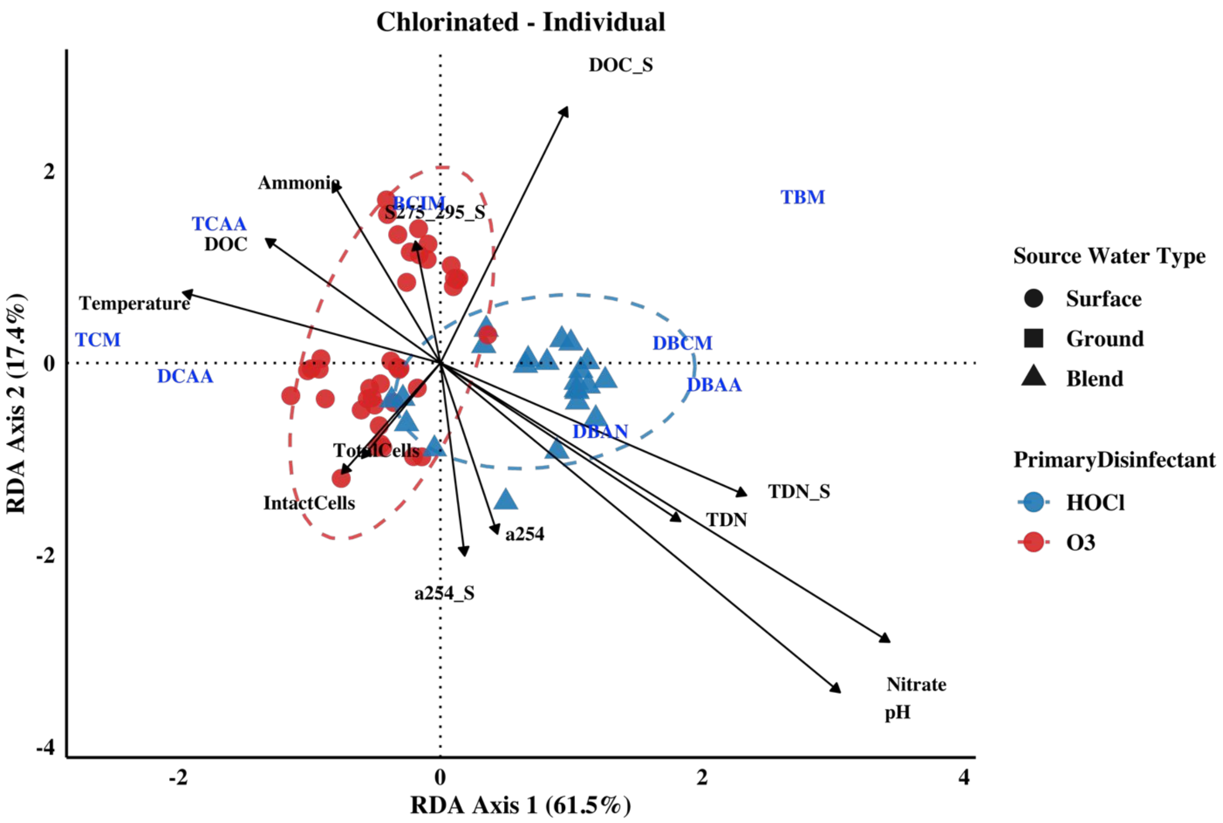

Figure SA4. RDA biplot of water quality parameters and individual DBP concentration in chlorinated systems.

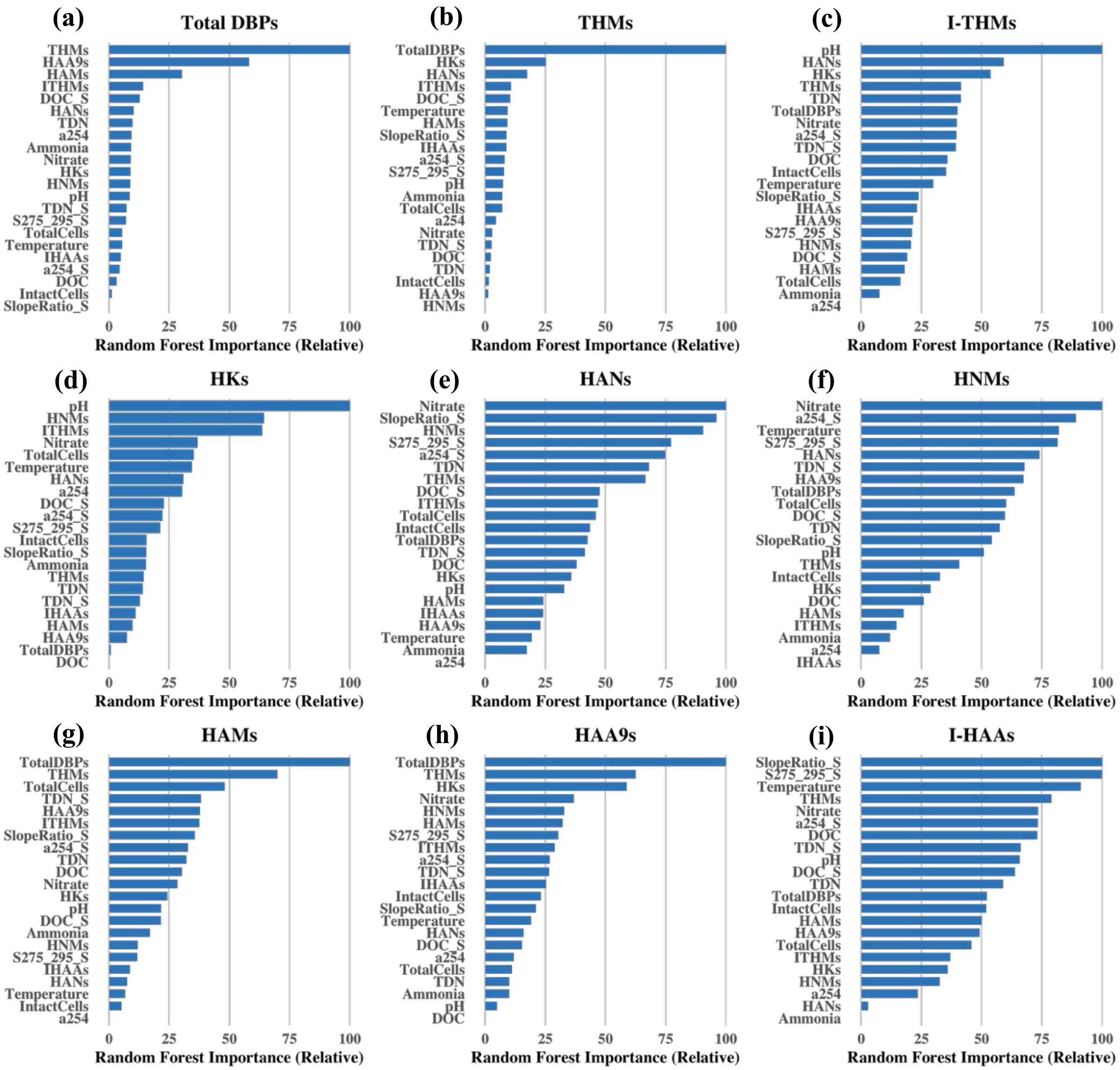

Figure SA5. Random Forest variable relative importance graphs for chlorinated systems for the concentrations of the following DBP classes: (a) Total DBPs, (b) THMs, (c) I-THMs, (d) HKs, (e) HANs, (f) HNMs, (g) HAMs, (h) HAA9s, and (i) I-HAAs.

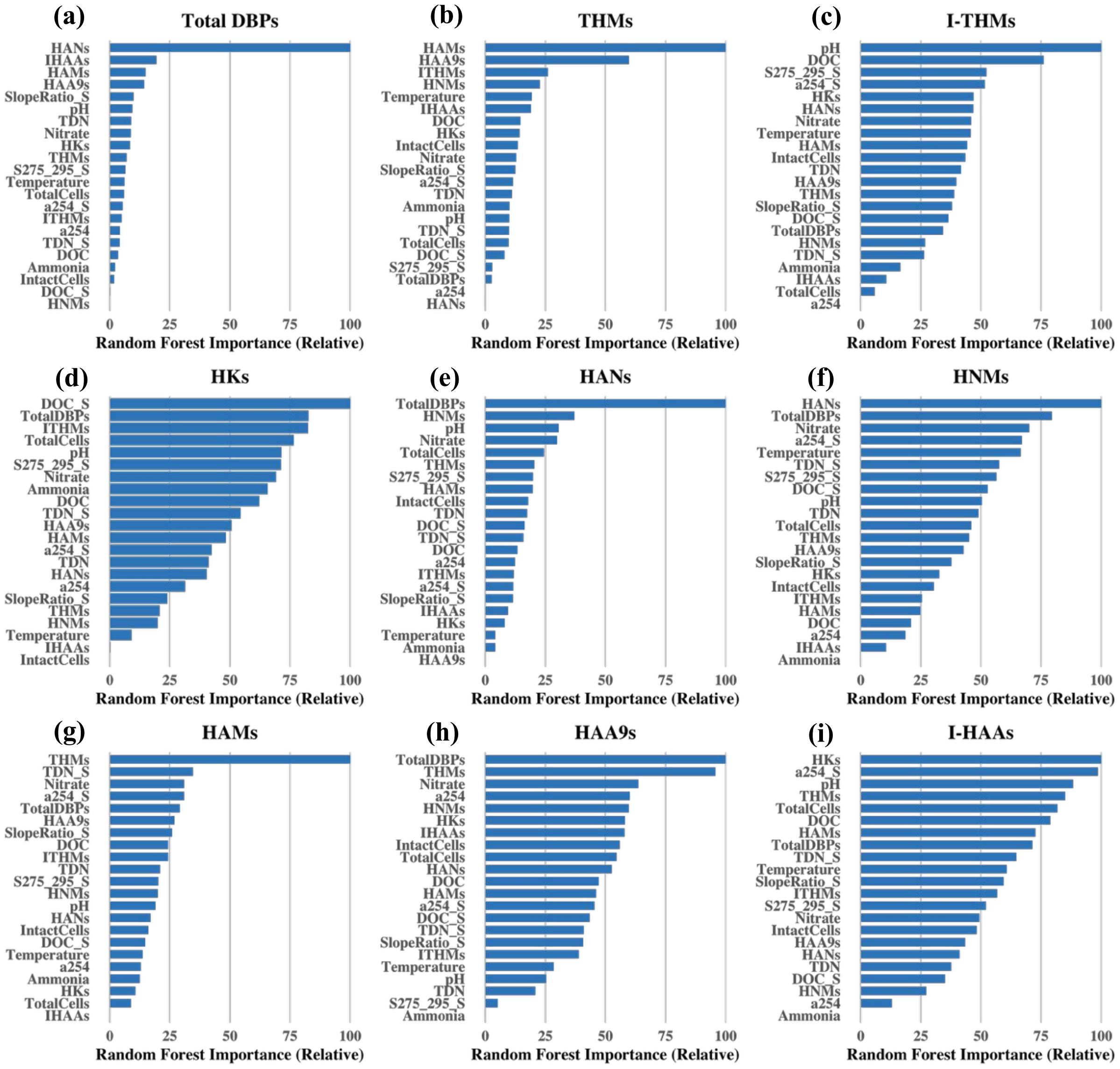

Figure SA6. Random Forest variable relative importance graphs for chlorinated systems for the cytotoxicity of the following DBP classes: (a) Total DBPs, (b) THMs, (c) I-THMs, (d) HKs, (e) HANs, (f) HNMs, (g) HAMs, (h) HAA9s, and (i) I-HAAs.

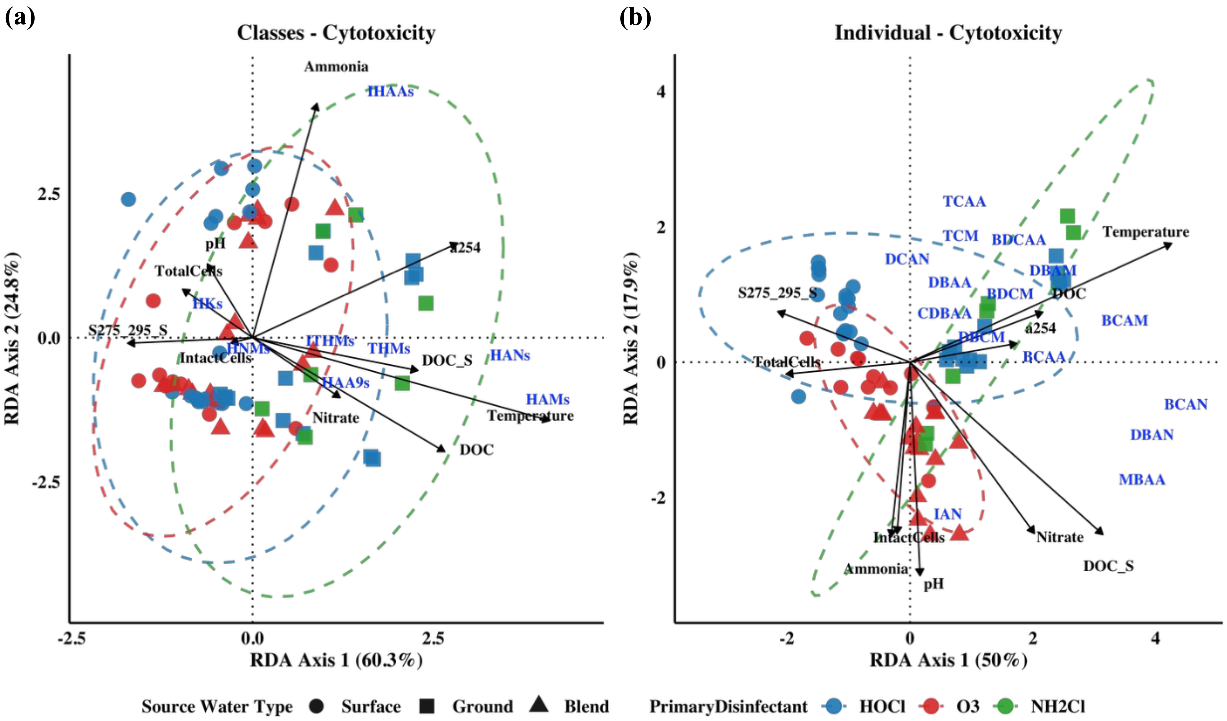

Figure SA7. RDA biplot of water quality parameters and DBP cytotoxicity in chloraminated systems grouped based on (a) classes of DBPs and (b) individual DBPs.

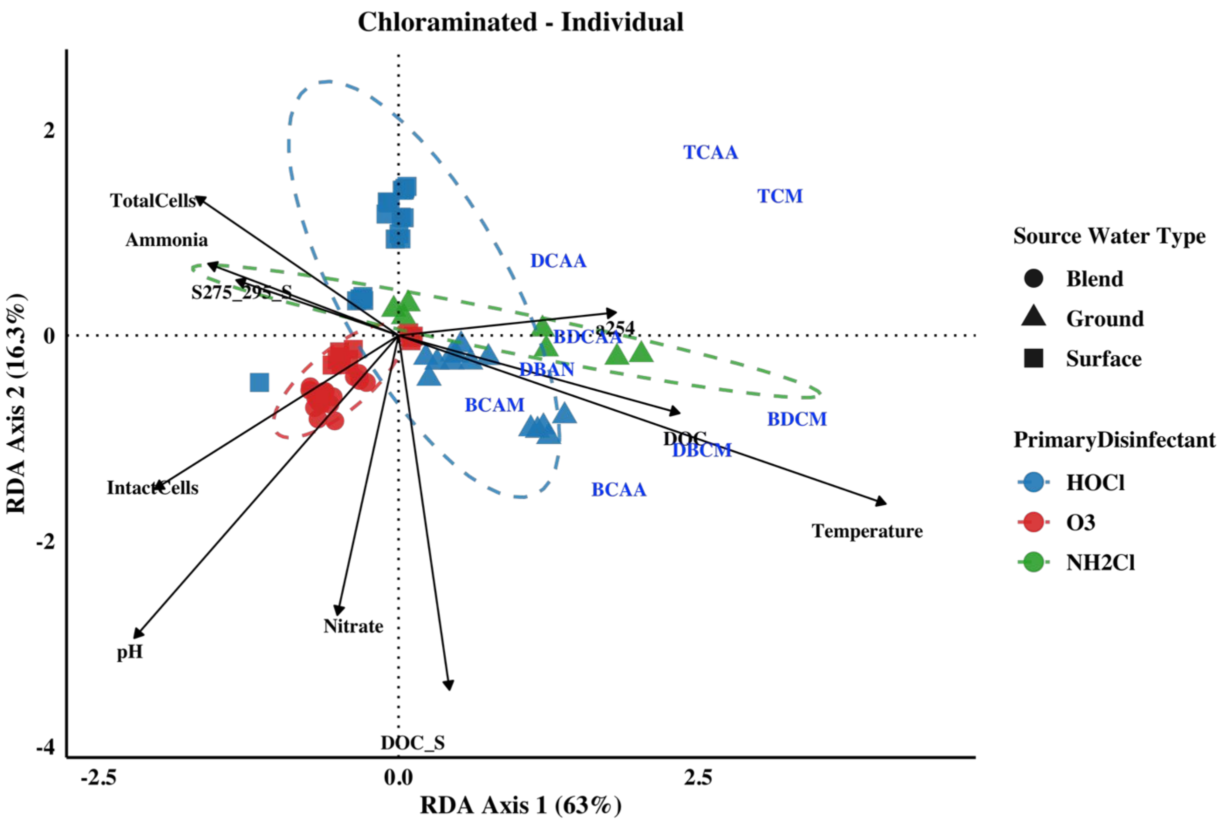

Figure SA8. RDA biplot of water quality parameters and individual DBP concentration in chloraminated systems.

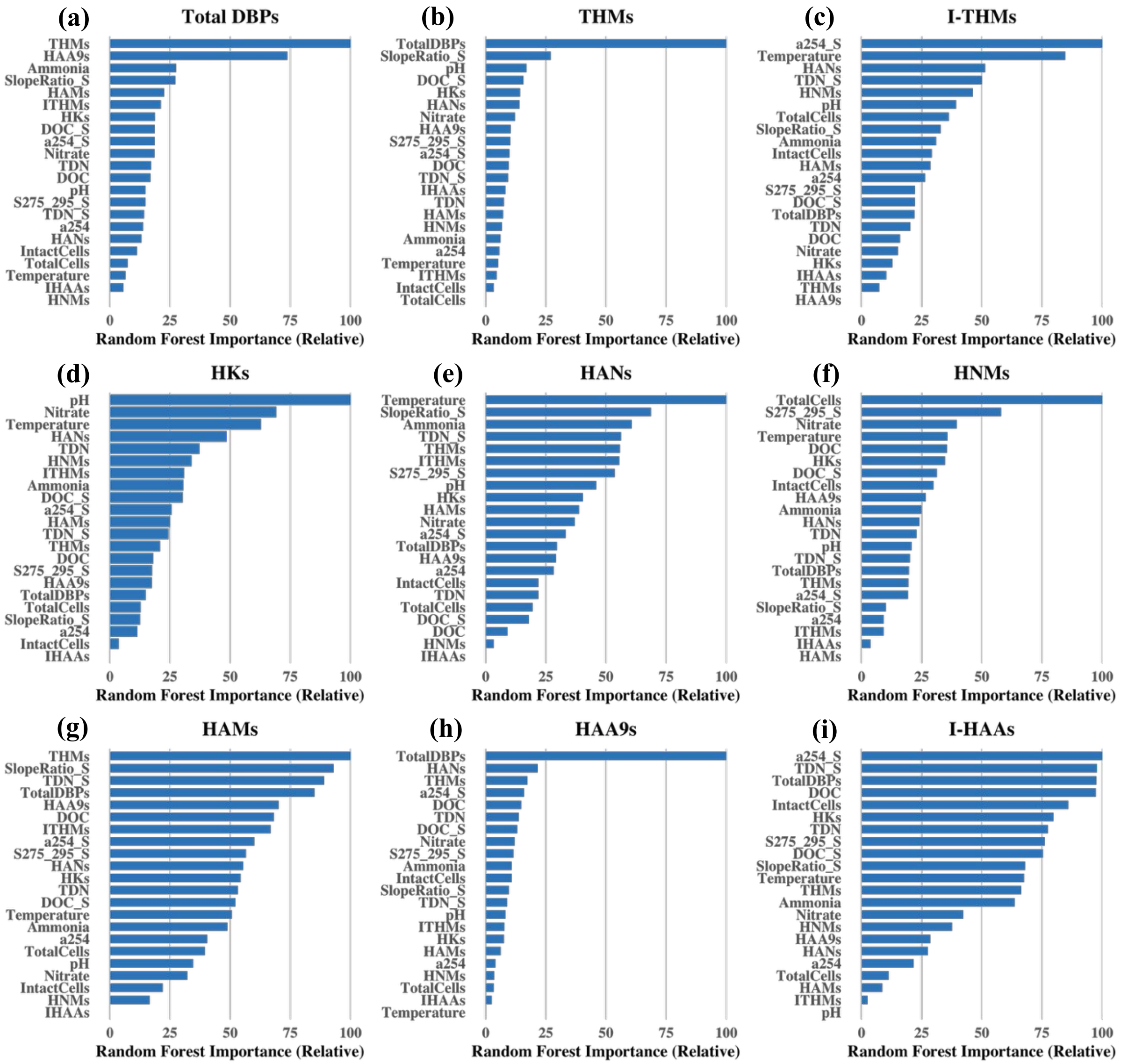

Figure SA9. Random Forest variable relative importance graphs for chloraminated systems for the concentrations of the following DBP classes: (a) Total DBPs, (b) THMs, (c) I-THMs, (d) HKs, (e) HANs, (f) HNMs, (g) HAMs, (h) HAA9s, and (i) I-HAAs.

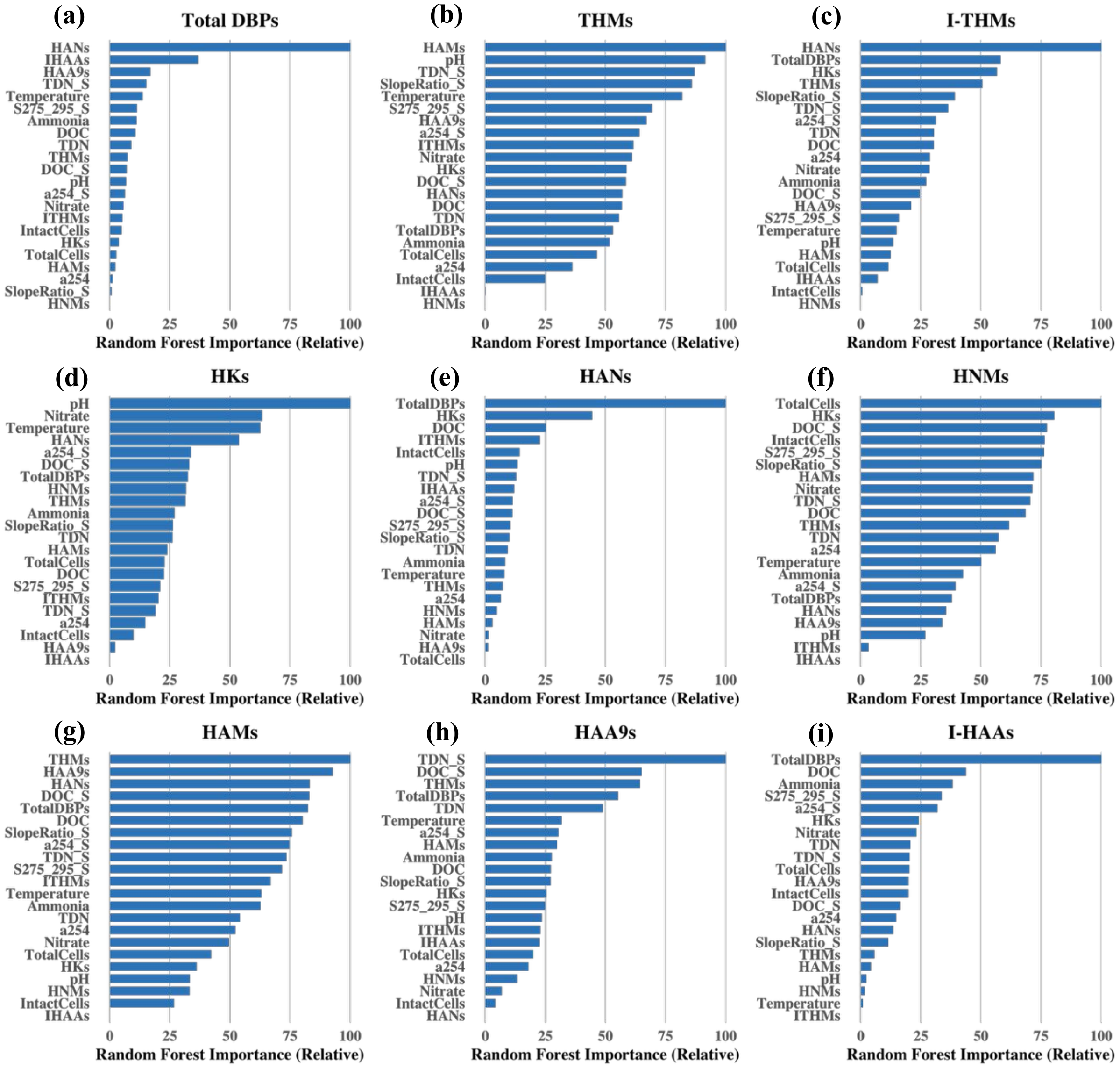

Figure SA10. Random Forest variable relative importance graphs for chloraminated systems for the cytotoxicity of the following DBP classes: (a) Total DBPs, (b) THMs, (c) I-THMs, (d) HKs, (e) HANs, (f) HNMs, (g) HAMs, (h) HAA9s, and (i) I-HAAs.

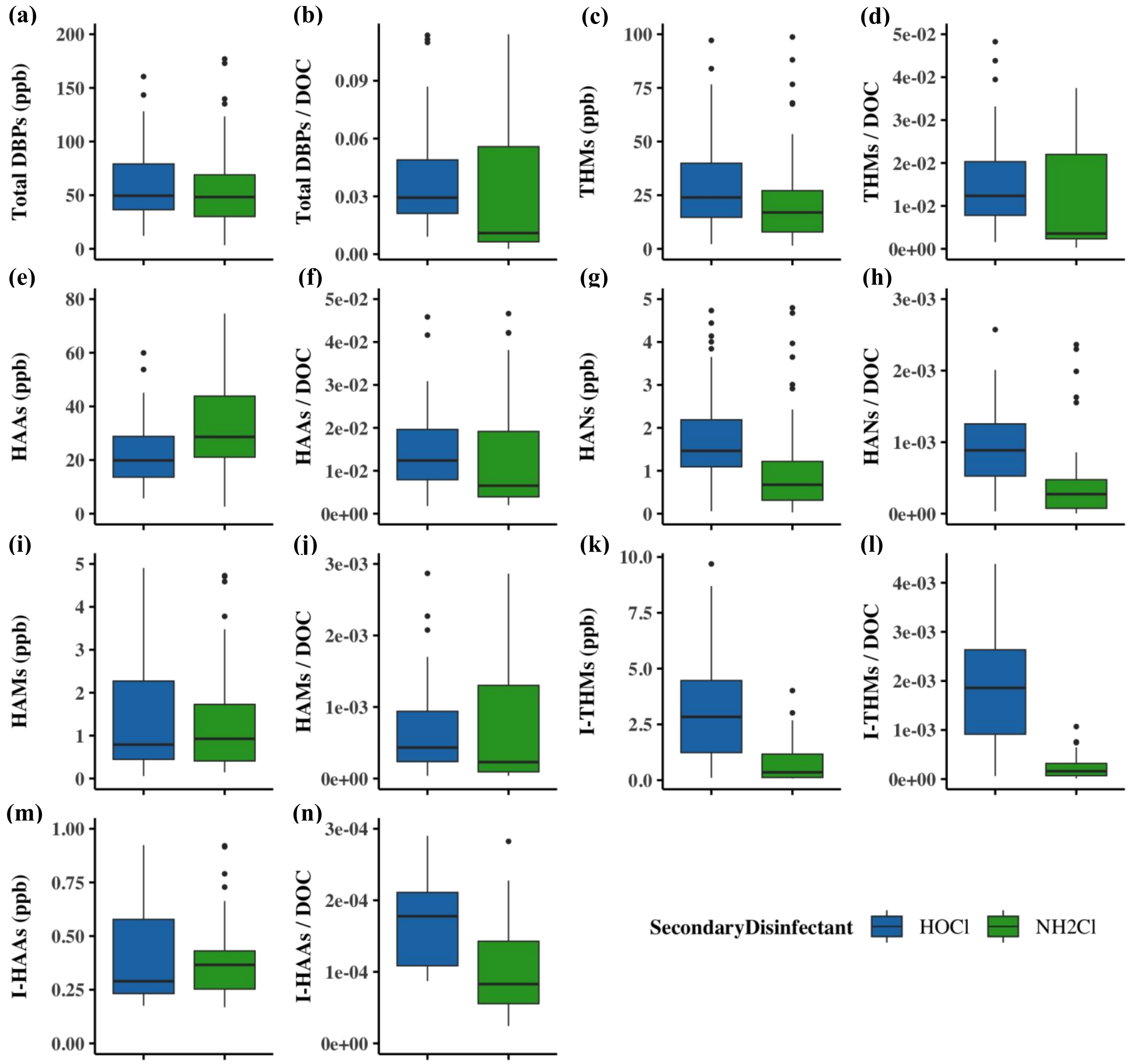

Figure SA11. Comparison between DBP concentrations and DOC-normalized concentrations for DBP classes by secondary disinfectant. (a) and (b) Total DBPs, (c) and (d) THMs, (e) and (f) HAAs, (g) and (h) HANs, (i) and (j) HAMs, (k) and (l) I-THMs, and (m) and (n) I-HAAs. Some outliers were removed for visualization. Classes with low concentrations were not included in this figure.

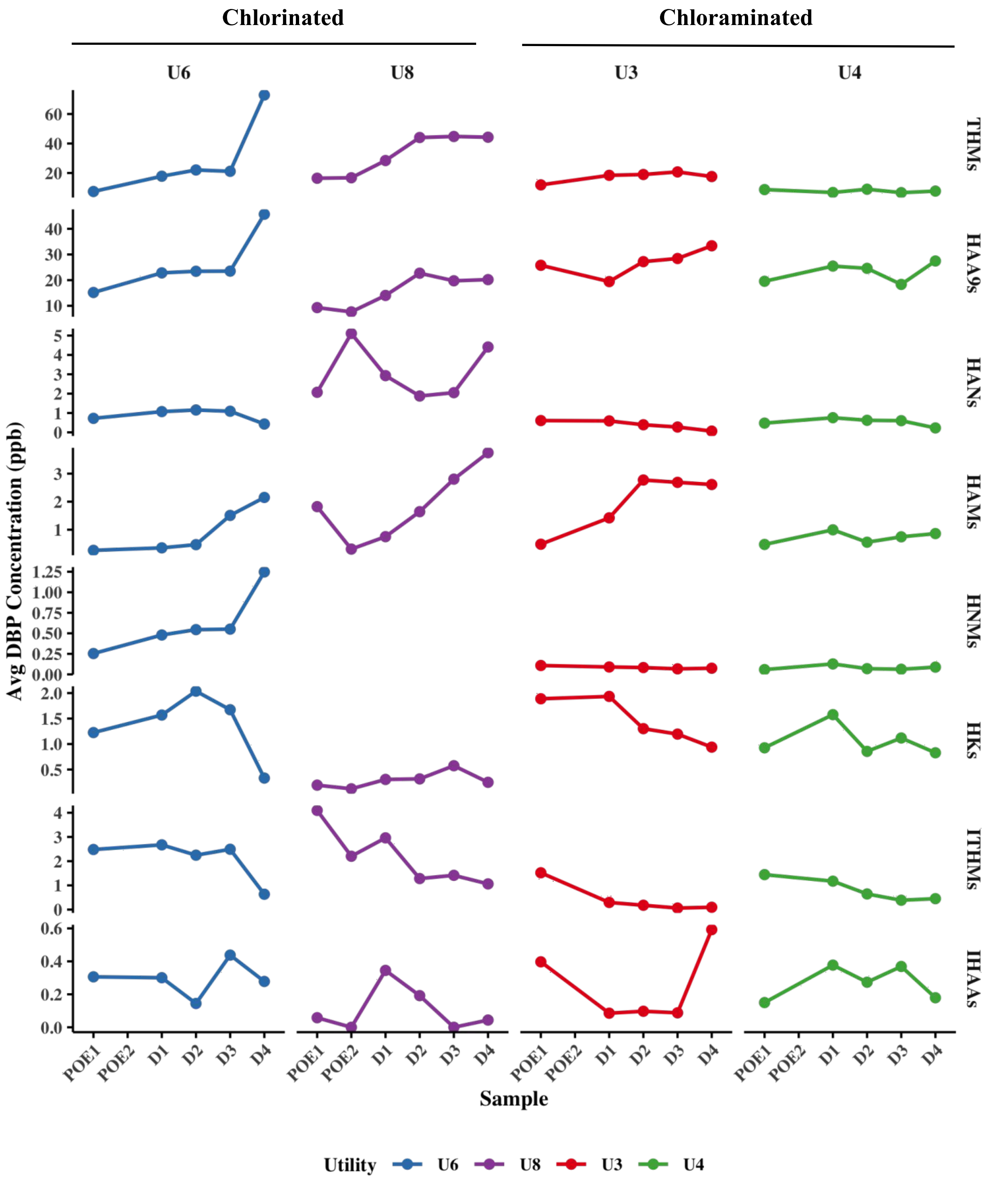

Figure SA12. Average DBP concentration across water age* (sample site used as proxy). * = Only utilities with provided water age data were included.

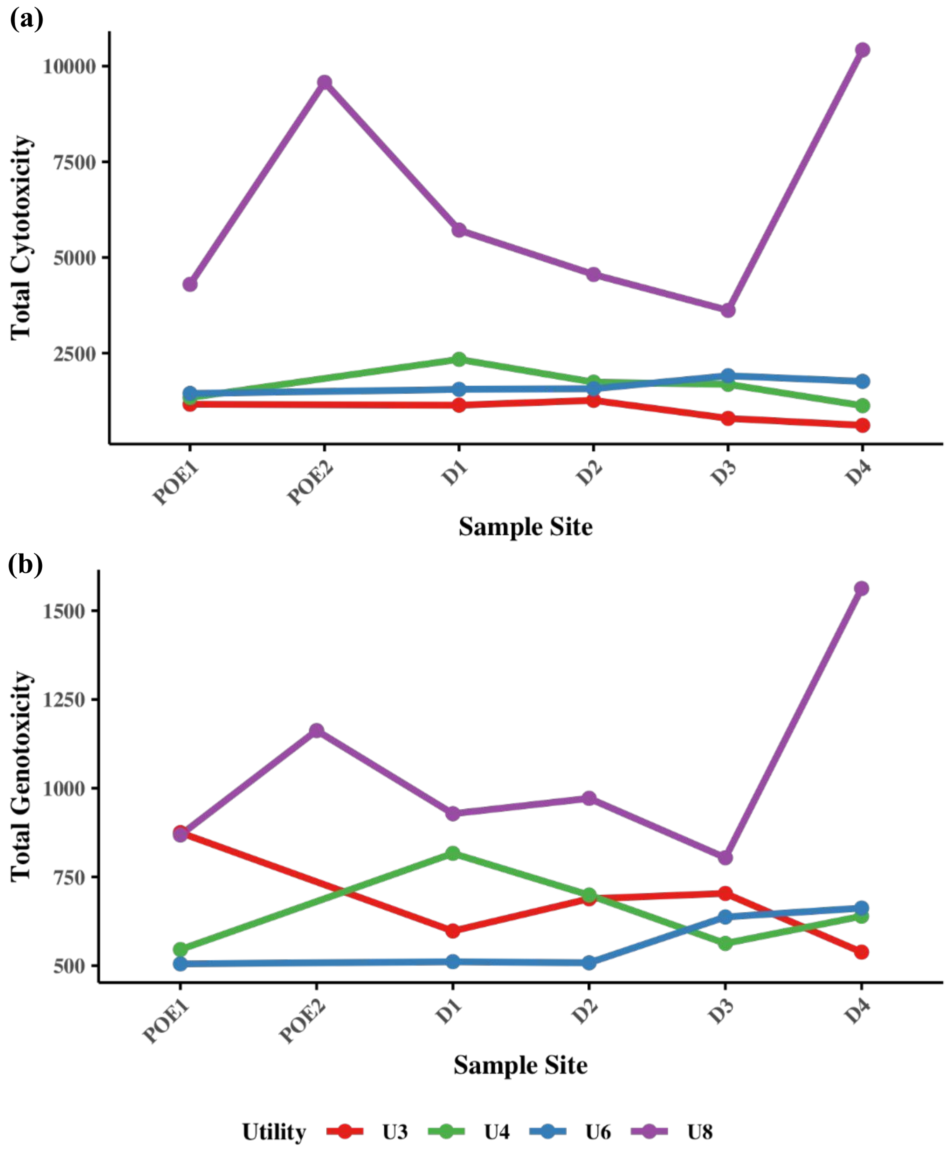

Figure SA13. Average total DBP (a) cytotoxicity and (b) genotoxicity across water age* (sample site used as proxy). Only utilities with provided water age data were included.

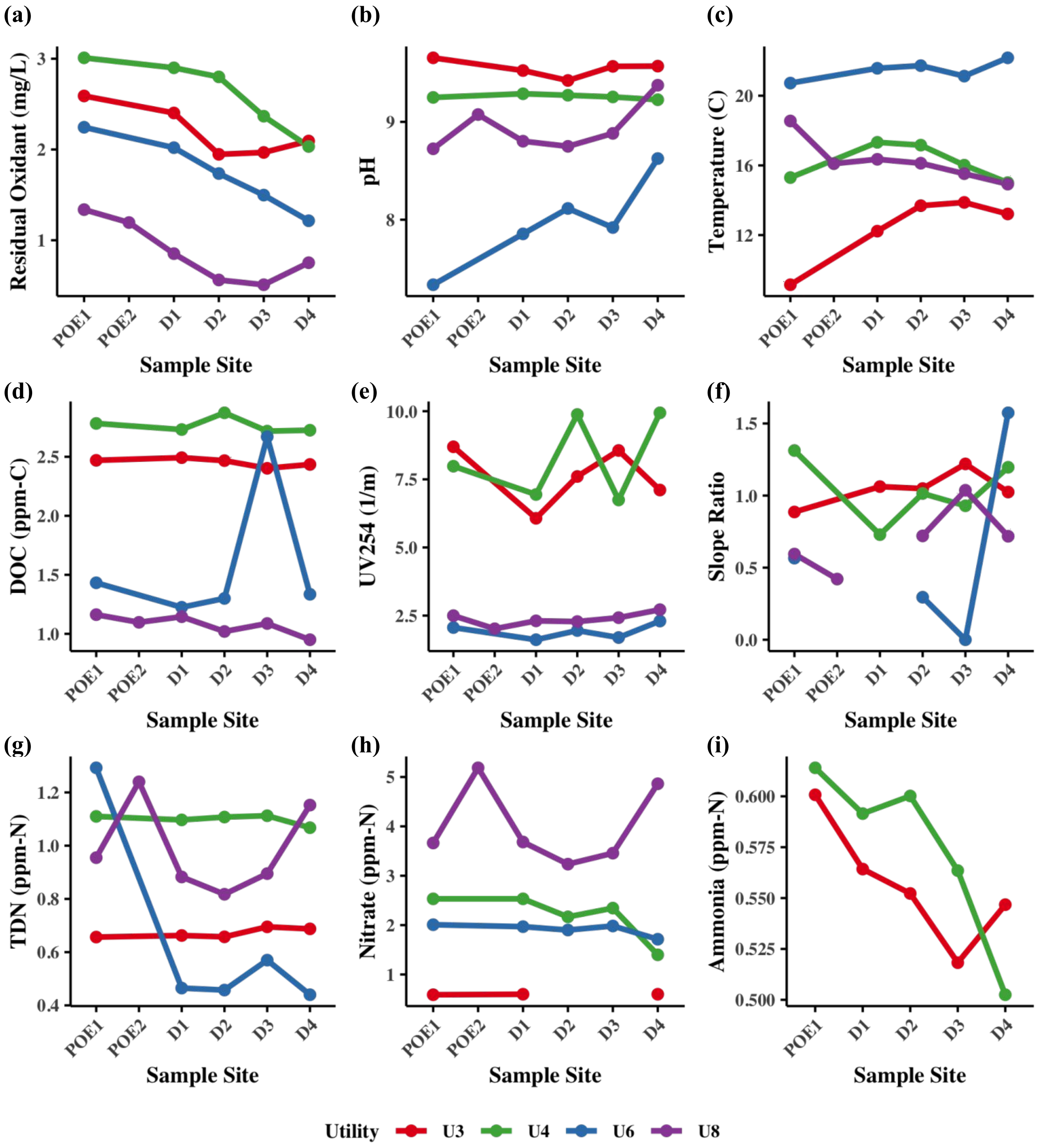

Figure SA14. (a) Residual oxidant (b) pH (c) temperature, (d) DOC, (e) UV_{254}, (f) slope ratio, (g) TDN, (h) nitrate, and (i) ammonia across sample site (proxy for water age*). Values averaged across sampling rounds. * = Only utilities with provided water age data were included.

References

(1) Li, J.; Arnold, W. A.; Hozalski, R. M. Spatiotemporal Variability in N-Nitrosodimethylamine Precursor Levels in a Watershed Impacted by Agricultural Activities and Municipal Wastewater Discharges and Effects of Lime Softening. *Environ. Sci. Technol.* **2023**, *57* (37), 13959–13969. https://doi.org/10.1021/acs.est.3c01767.

(2) Stubbins, A.; Dittmar, T. Low Volume Quantification of Dissolved Organic Carbon and Dissolved Nitrogen. *Limnol. Oceanogr. Methods* **2012**, *10* (5), 347–352. https://doi.org/10.4319/lom.2012.10.347.

(3) Stubbins, A.; Law, C. S.; Uher, G.; Upstill-Goddard, R. C. Carbon Monoxide Apparent Quantum Yields and Photoproduction in the Tyne Estuary. *Biogeosciences* **2011**, *8* (3), 703–713. https://doi.org/10.5194/bg-8-703-2011.

(4) Hu, C.; Muller-Karger, F. E.; Zepp, R. G. Absorbance, Absorption Coefficient, and Apparent Quantum Yield: A Comment on Common Ambiguity in the Use of These Optical Concepts. *Limnol. Oceanogr.* **2002**, *47* (4), 1261–1267. https://doi.org/10.4319/lo.2002.47.4.1261.

(5) Weishaar, J. L.; Aiken, G. R.; Bergamaschi, B. A.; Fram, M. S.; Fujii, R.; Mopper, K. Evaluation of Specific Ultraviolet Absorbance as an Indicator of the Chemical Composition and Reactivity of Dissolved Organic Carbon. *Environ. Sci. Technol.* **2003**, *37* (20), 4702–4708. https://doi.org/10.1021/es030360x.

(6) Helms, J. R.; Stubbins, A.; Ritchie, J. D.; Minor, E. C.; Kieber, D. J.; Mopper, K. Absorption Spectral Slopes and Slope Ratios as Indicators of Molecular Weight, Source, and Photobleaching of Chromophoric Dissolved Organic Matter. *Limnol. Oceanogr.* **2008**, *53* (3), 955–969. https://doi.org/10.4319/lo.2008.53.3.0955.
