# Supplementary material for: Identification of Factors Influencing Variability in Disinfection Byproducts and Their Toxicity in Chlorinated and Chloraminated Drinking Water Distribution Systems across the United States
Source: Environ Sci Technol. 2025 Dec 31;60(1):1241–52. doi: 10.1021/acs.est.5c12121 (PMC12810234; doi:10.1021/acs.est.5c12121)
Supplement: Supplementary file 2 [file es5c12121_si_002.pdf]

Supporting Information B

Identification of factors influencing variability in disinfection byproducts and their toxicity in chlorinated and chloraminated drinking water distribution systems across the United States

Samantha DiLoreto[1]; Huanqi He[1,2]; Jinhao Yang[1]; Patrick Milne[3]; Jiaqi Li[1]; Christopher A. Impellitteri[4]; Aron Stubbins[3,5,6]; Ameet Pinto[1,7]; Ching-Hua Huang[1,*]

[1] School of Civil and Environmental Engineering, Georgia Institute of Technology, Atlanta, Georgia, 30332, USA

[2] School of Science and Engineering, Benedict College, Columbia, South Carolina, 29204, USA

[3] Department of Chemistry and Chemical Biology, Northeastern University, Boston, Massachusetts, 02115, USA

[4] The Water Tower, Buford, Georgia, 30519, USA

[5] Department of Marine and Environmental Sciences, Northeastern University, Boston, Massachusetts, 02115, USA

[6] Department of Civil and Environmental Engineering, Northeastern University, Boston, Massachusetts, 02115, USA

[7] School of Earth and Atmospheric Sciences, Georgia Institute of Technology, Atlanta, Georgia, 30332, USA

* Corresponding author: Ching-Hua Huang (ching-hua.huang@ce.gatech.edu)

Table of Contents

Figure SB1. Spearman correlations for (a) DBP concentrations and (b) DBP cytotoxicity with water quality parameters and microbial cell counts in chlorinated systems.

Figure SB2. Spearman correlations for DBP concentrations, water quality parameters, and microbial cell counts at individual chlorinated utilities (a) U2, (b) U6, (c) U8.

Figure SB3. Spearman correlations for DBP cytotoxicity, water quality parameters, and microbial cell counts at individual chlorinated utilities (a) U2, (b) U6, (c) U8.

Figure SB4. Spearman correlations for (a) DBP concentrations and (b) DBP cytotoxicity with water quality parameters and microbial cell counts in chloraminated systems.

Figure SB5. Spearman correlations for DBP concentrations, water quality parameters, and microbial cell counts at individual chloraminated utilities (a) U3, (b) U4, (c) U5, (d) U7, and (e) U9.

Figure SB6. Spearman correlations for DBP cytotoxicity, water quality parameters, and microbial cell counts at individual chloraminated utilities (a) U3, (b) U4, (c) U5, (d) U7, and (e) U9.

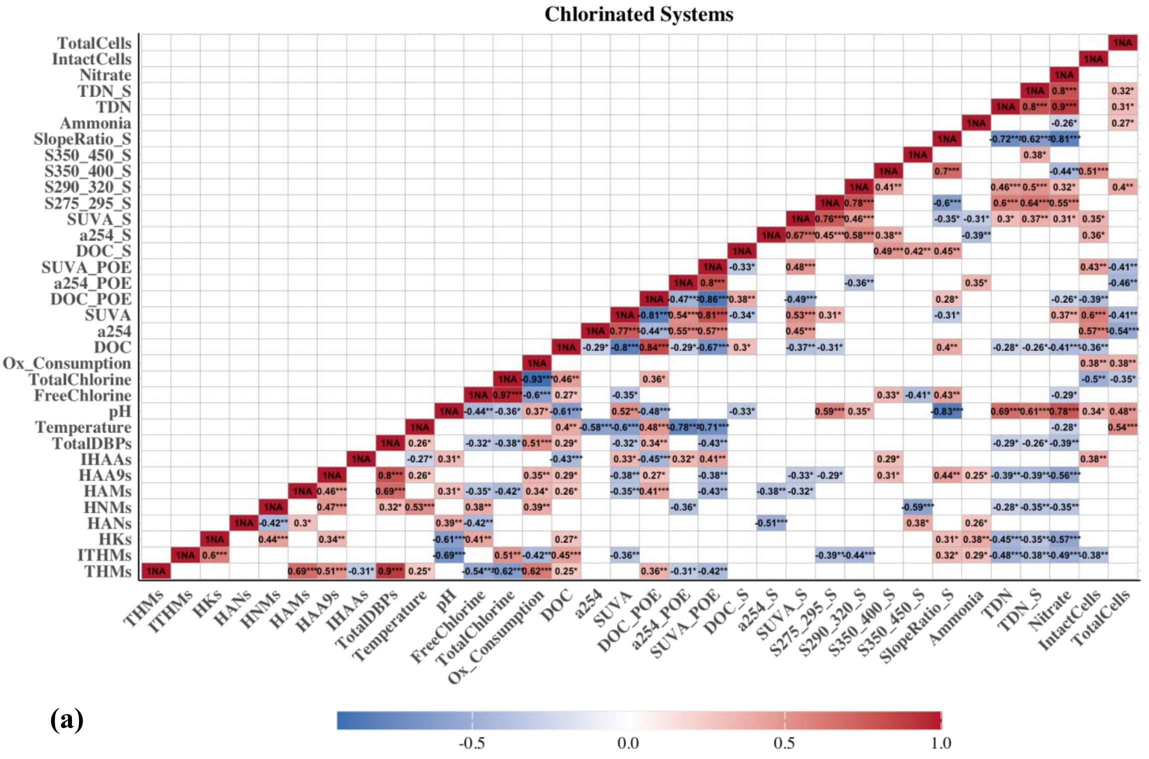

(a)

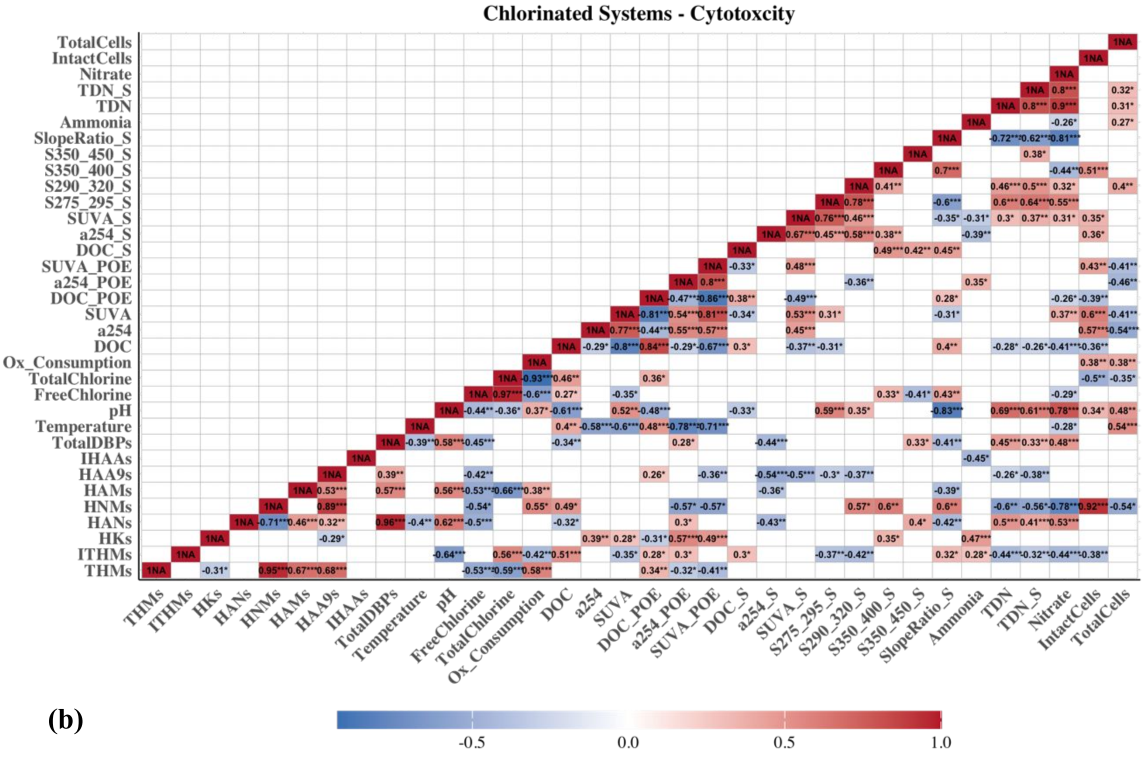

(b)

Figure SB1. Spearman correlations for (a) DBP concentrations and (b) DBP cytotoxicity with water quality parameters and microbial cell counts in chlorinated systems.

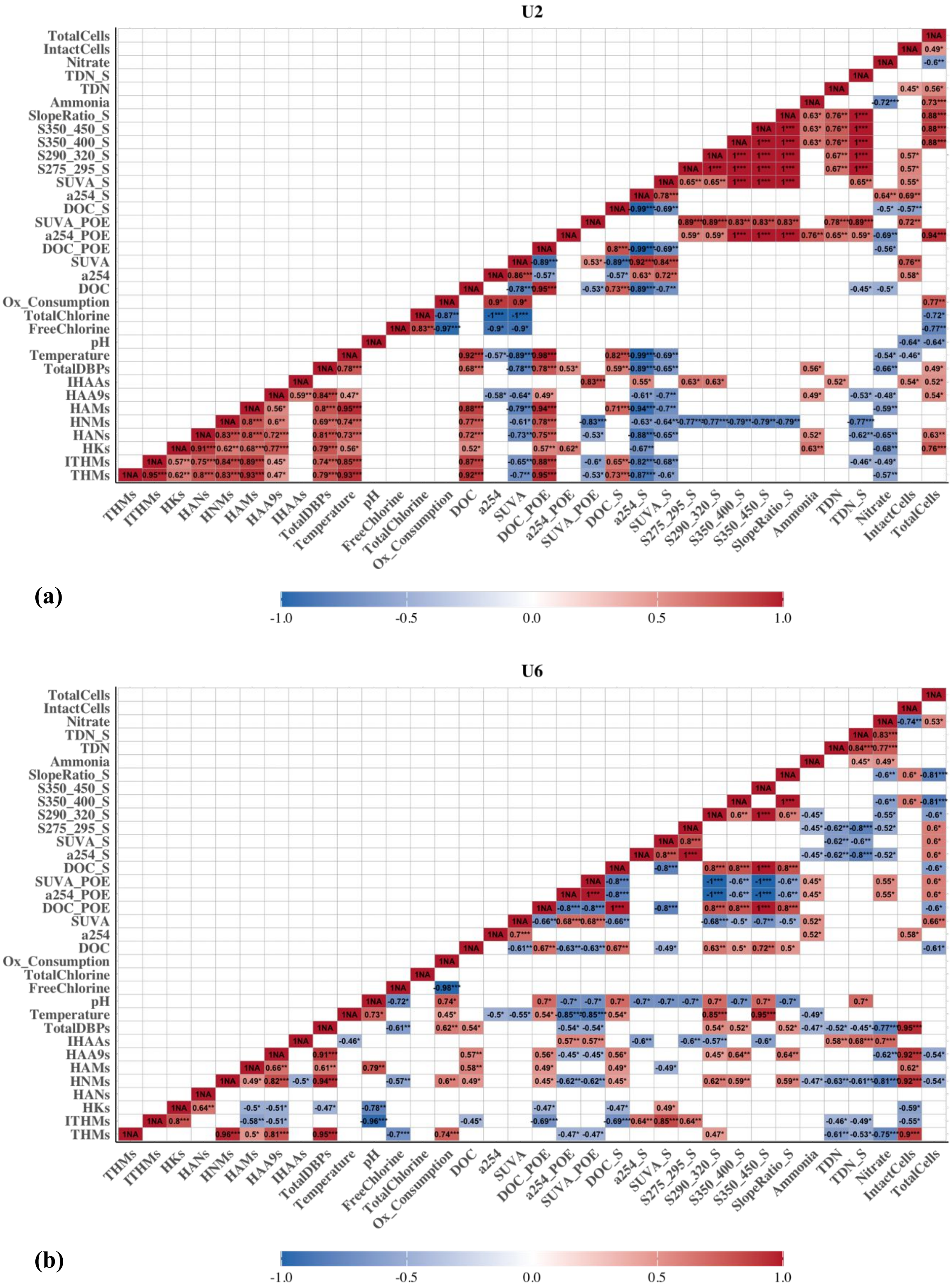
U2
TotalCells
IntactCells
Nitrate
TDN_S
TDN
Ammonia
SlopeRatio_S
S350_450_S
S350_400_S
S290_320_S
S275_295_S
SUVA_S
a254_S
DOC_S
SUVA_POE
a254_POE
DOC_POE
SUVA
a254
DOC
Ox_Consumption
TotalChlorine
FreeChlorine
pH
Temperature
TotalDBPs
IHAAs
HAA9s
HAMs
HNMs
HANs
HKs
ITHMs
THMs
(a)
-1.0
-0.5
0.0
0.5
1.0
U6
(b)

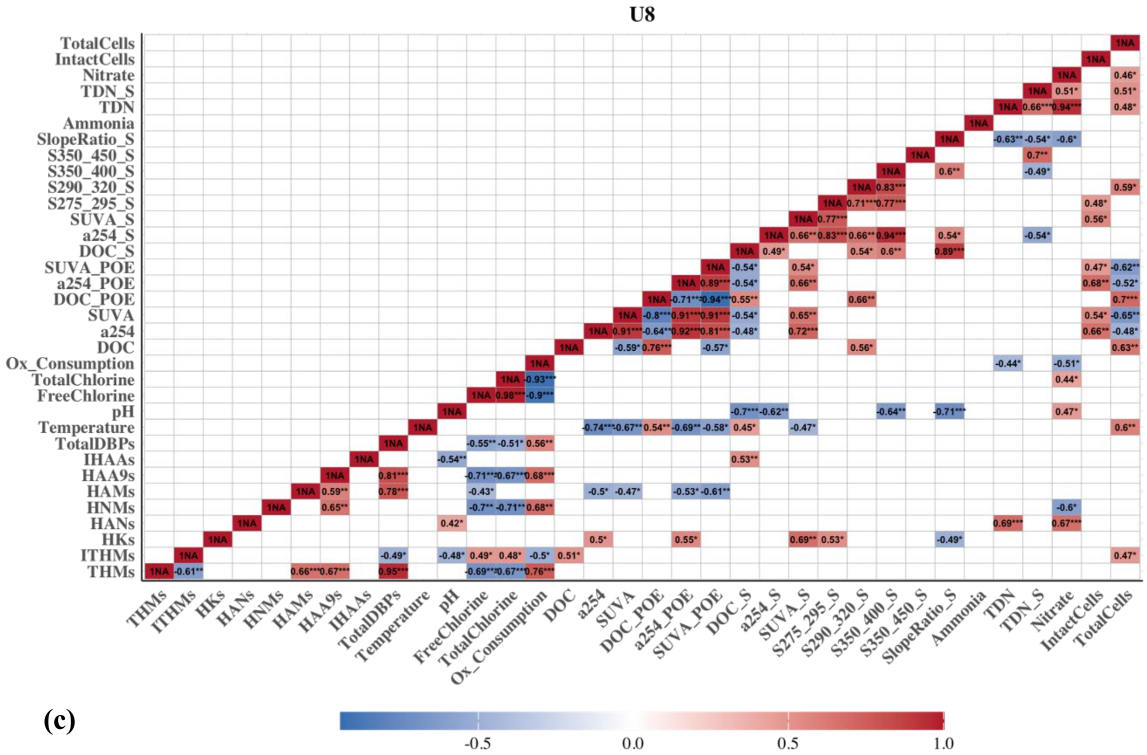

(c)

Figure SB2. Spearman correlations for DBP concentrations, water quality parameters, and microbial cell counts at individual chlorinated utilities (a) U2, (b) U6, (c) U8.

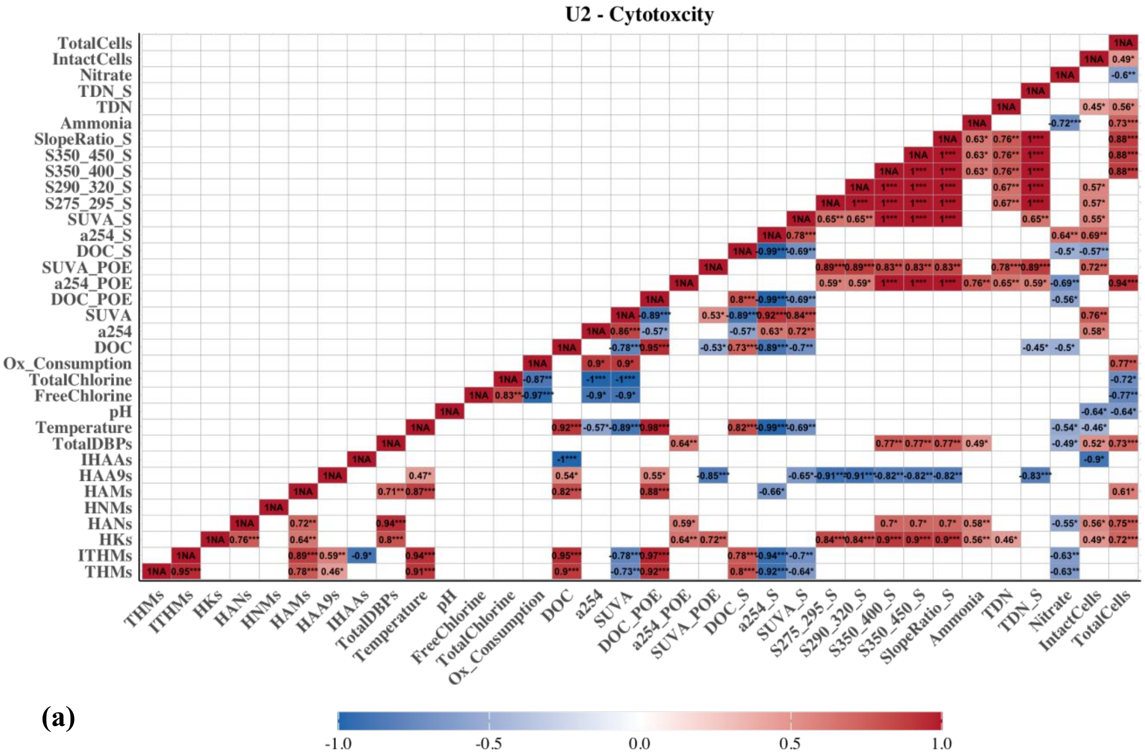
U2 - Cytotoxcity
TotalCells
IntactCells
Nitrate
TDN_S
TDN
Ammonia
SlopeRatio_S
S350_450_S
S350_400_S
S290_320_S
S275_295_S
SUVA_S
a254_S
DOC_S
SUVA_POE
a254_POE
DOC_POE
SUVA
a254
DOC
Ox_Consumption
TotalChlorine
FreeChlorine
pH
Temperature
TotalDBPs
IHAAs
HAA9s
HAMs
HNMs
HANs
HKs
ITHMs
THMs
-1.0
-0.5
0.0
0.5
1.0

(a)

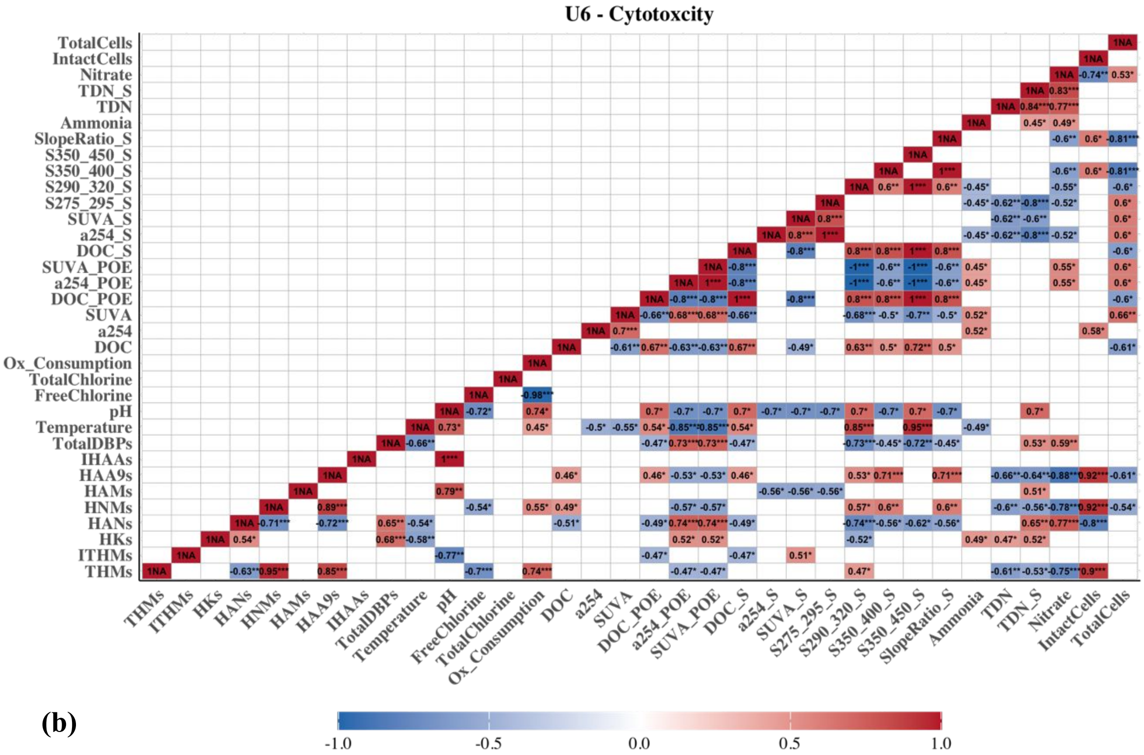
U6 - Cytotoxcity
TotalCells
IntactCells
Nitrate
TDN_S
TDN
Ammonia
SlopeRatio_S
S350_450_S
S350_400_S
S290_320_S
S275_295_S
SUVA_S
a254_S
DOC_S
SUVA_POE
a254_POE
DOC_POE
SUVA
a254
DOC
Ox_Consumption
TotalChlorine
FreeChlorine
pH
Temperature
TotalDBPs
IHAAs
HAA9s
HAMs
HNMs
HANs
HKs
ITHMs
THMs
-1.0
-0.5
0.0
0.5
1.0

(b)

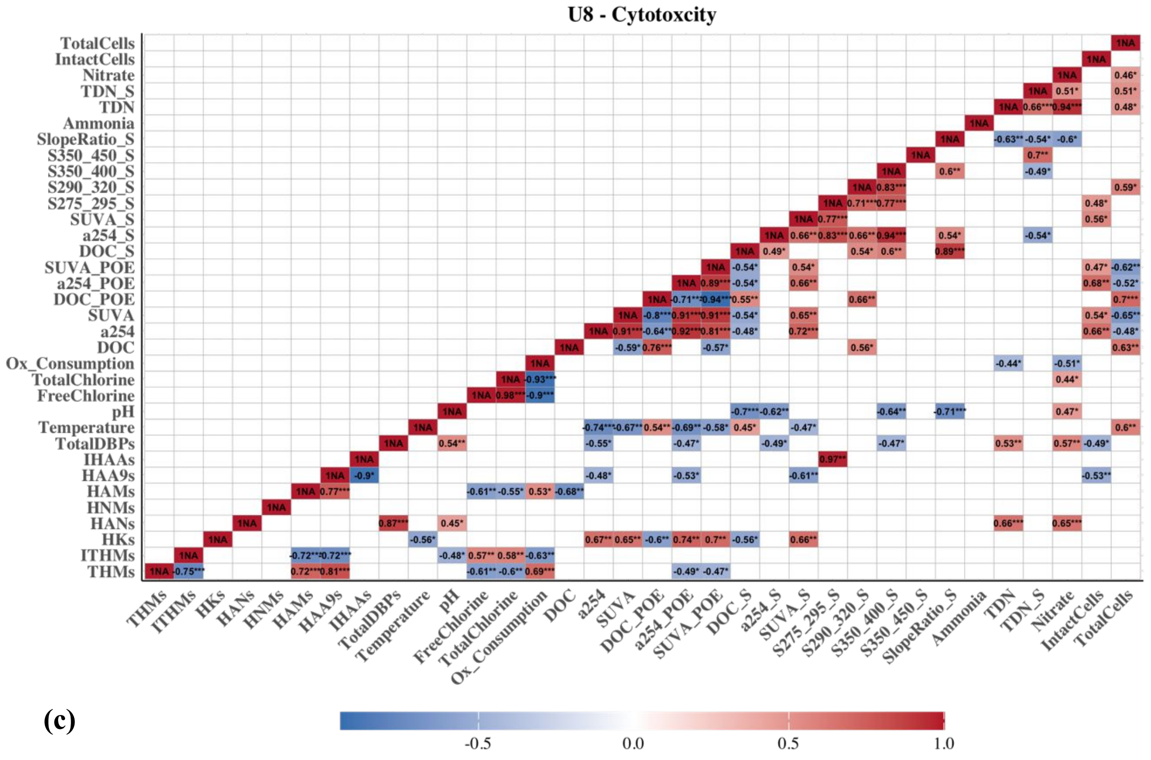

(c)

Figure SB3. Spearman correlations for DBP cytotoxicity, water quality parameters, and microbial cell counts at individual chlorinated utilities (a) U2, (b) U6, (c) U8.

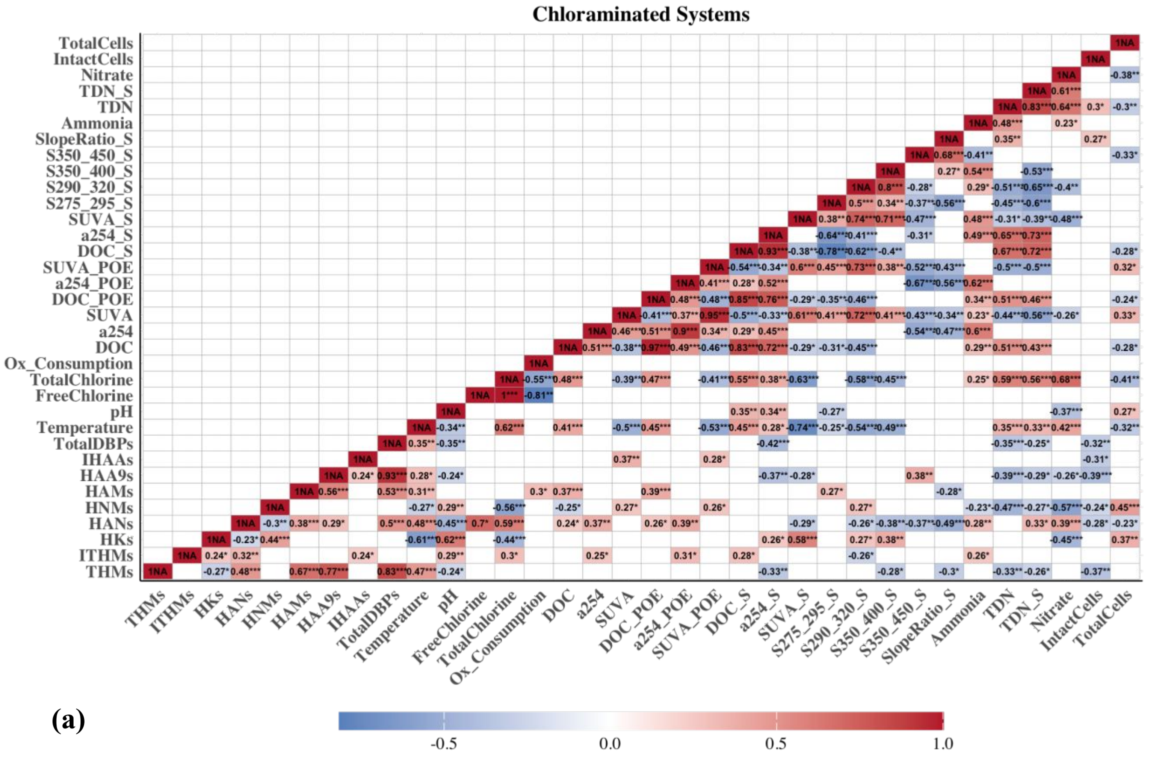

(a)

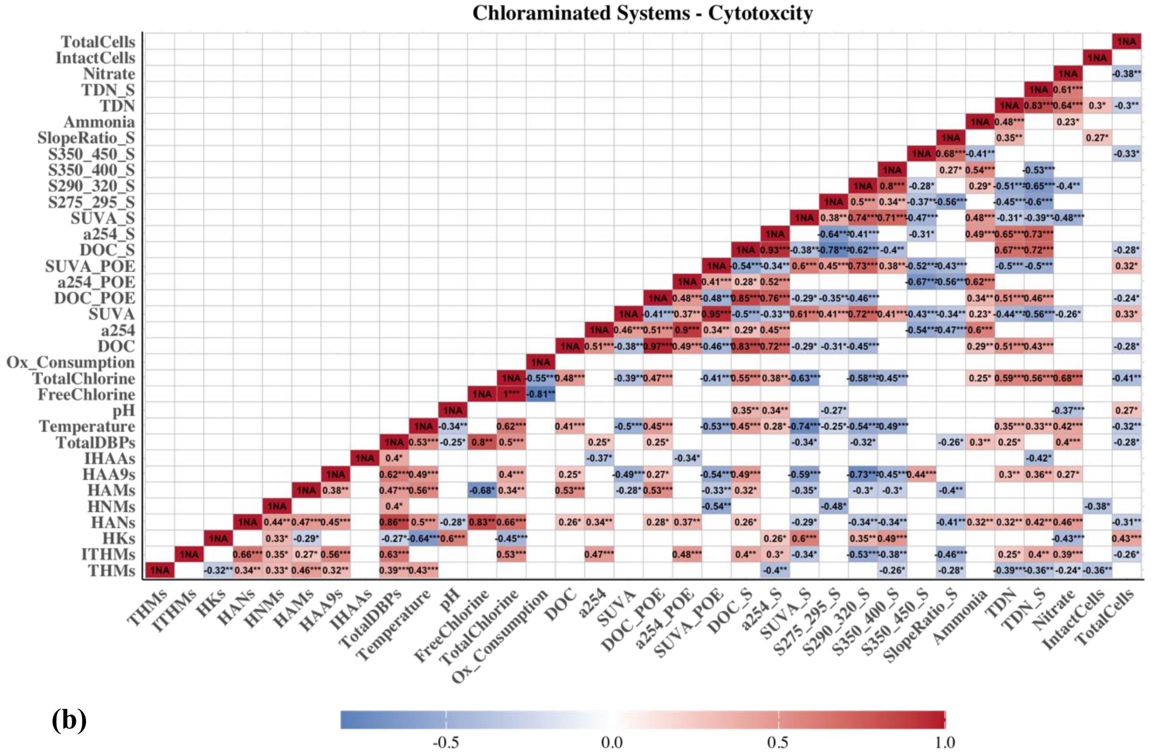

(b)

Figure SB4. Spearman correlations for (a) DBP concentrations and (b) DBP cytotoxicity with water quality parameters and microbial cell counts in chloraminated systems.

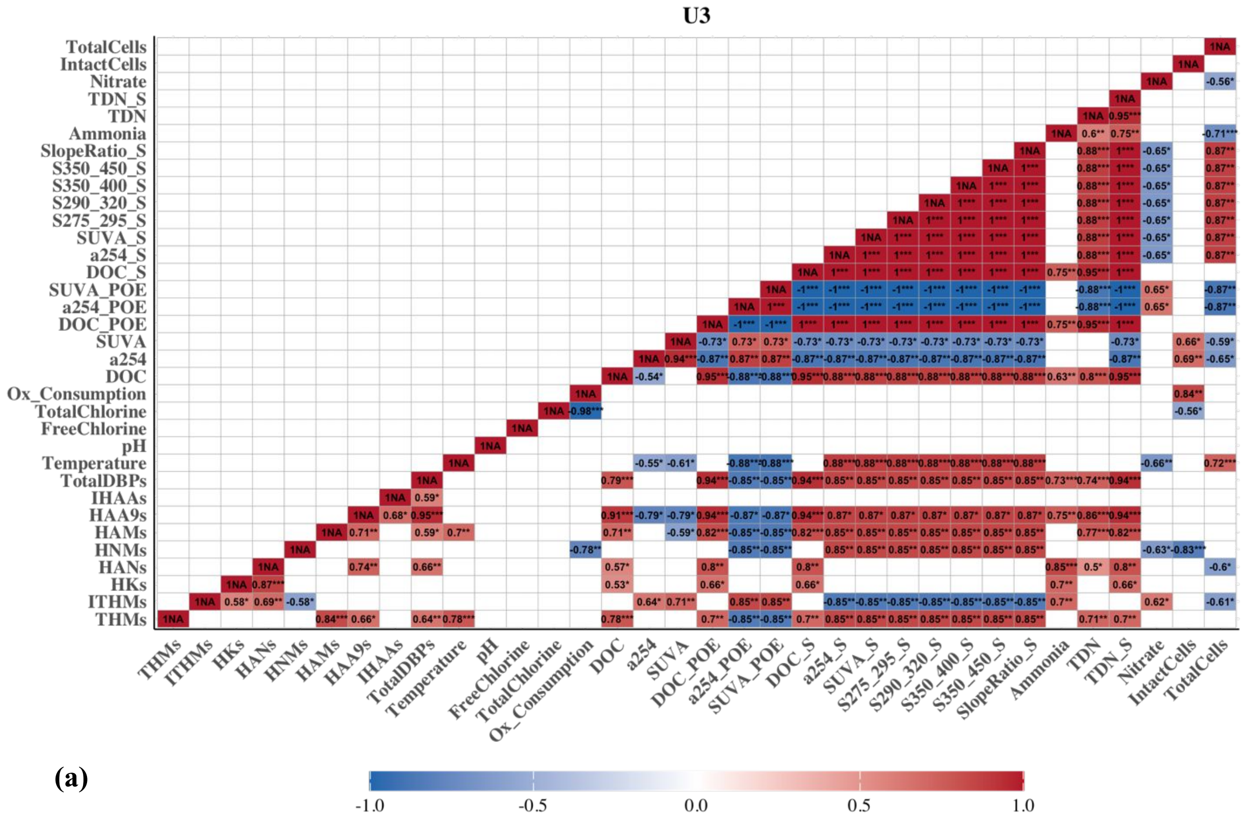
U3
TotalCells
IntactCells
Nitrate
TDN_S
TDN
Ammonia
SlopeRatio_S
S350_450_S
S350_400_S
S290_320_S
S275_295_S
SUVA_S
a254_S
DOC_S
SUVA_POE
a254_POE
DOC_POE
SUVA
a254
DOC
Ox_Consumption
TotalChlorine
FreeChlorine
pH
Temperature
TotalDBPs
IHAAs
HAA9s
HAMs
HNMs
HANs
HKs
ITHMs
THMs
-1.0
-0.5
0.0
0.5
1.0

(a)

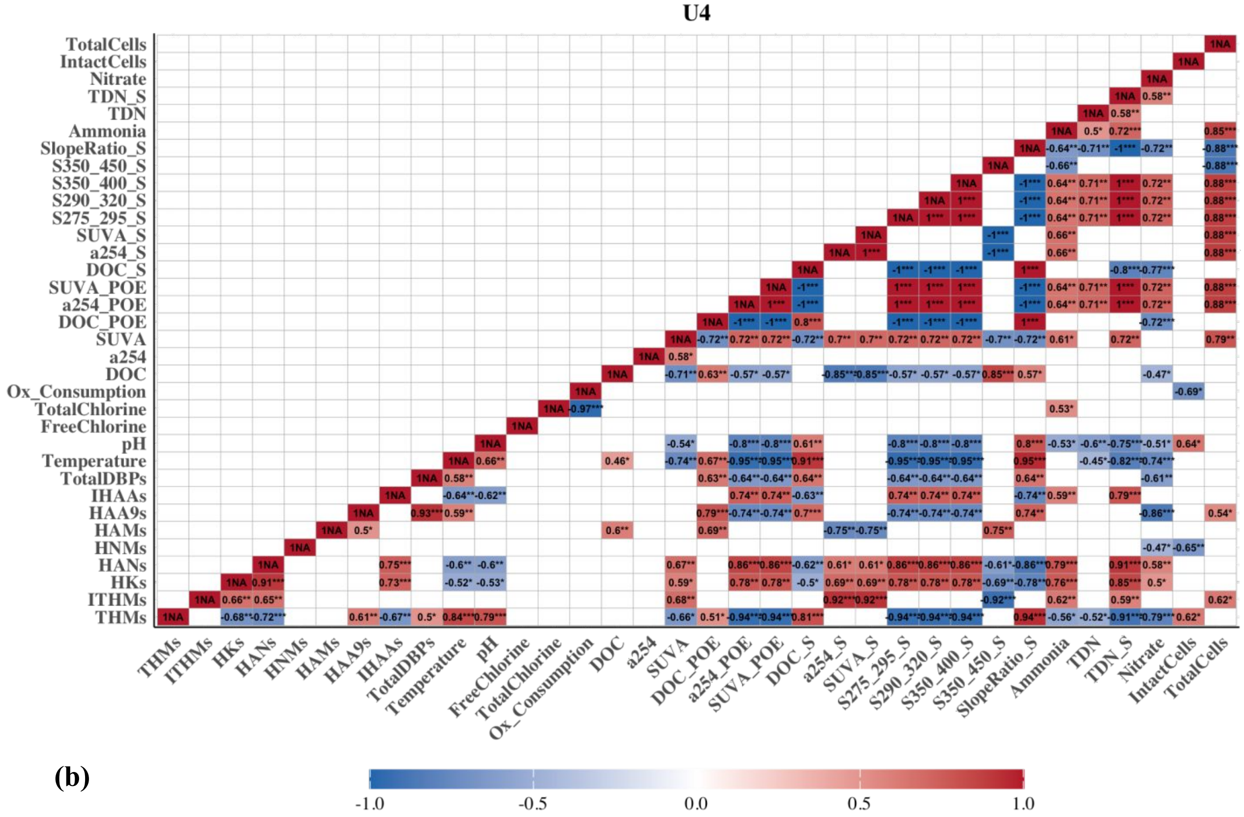
U4
TotalCells
IntactCells
Nitrate
TDN_S
TDN
Ammonia
SlopeRatio_S
S350_450_S
S350_400_S
S290_320_S
S275_295_S
SUVA_S
a254_S
DOC_S
SUVA_POE
a254_POE
DOC_POE
SUVA
a254
DOC
Ox_Consumption
TotalChlorine
FreeChlorine
pH
Temperature
TotalDBPs
IHAAs
HAA9s
HAMs
HNMs
HANs
HKs
ITHMs
THMs
-1.0
-0.5
0.0
0.5
1.0

(b)

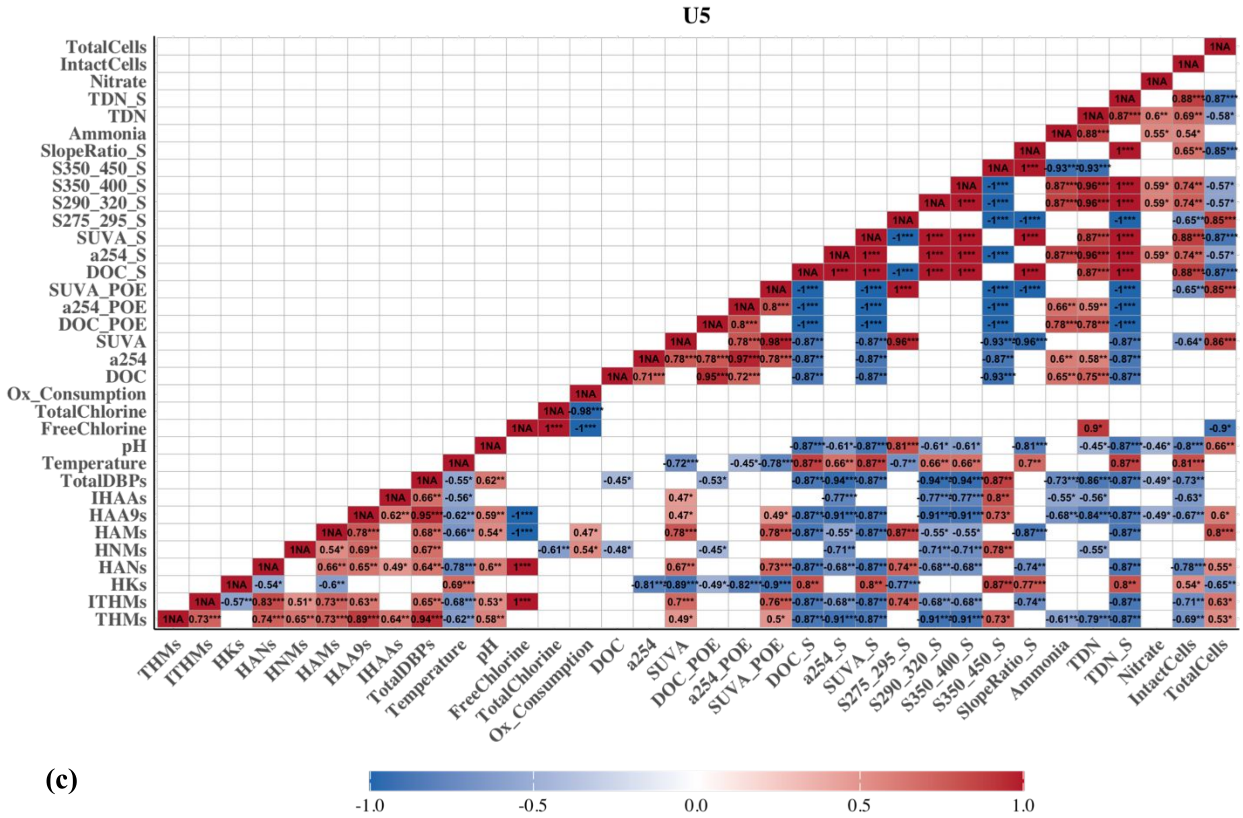
U5
TotalCells
IntactCells
Nitrate
TDN_S
TDN
Ammonia
SlopeRatio_S
S350_450_S
S350_400_S
S290_320_S
S275_295_S
SUVA_S
a254_S
DOC_S
SUVA_POE
a254_POE
DOC_POE
SUVA
a254
DOC
Ox_Consumption
TotalChlorine
FreeChlorine
pH
Temperature
TotalDBPs
IHAAs
HAA9s
HAMs
HNMs
HANs
HKs
ITHMs
THMs
-1.0
-0.5
0.0
0.5
1.0

(c)

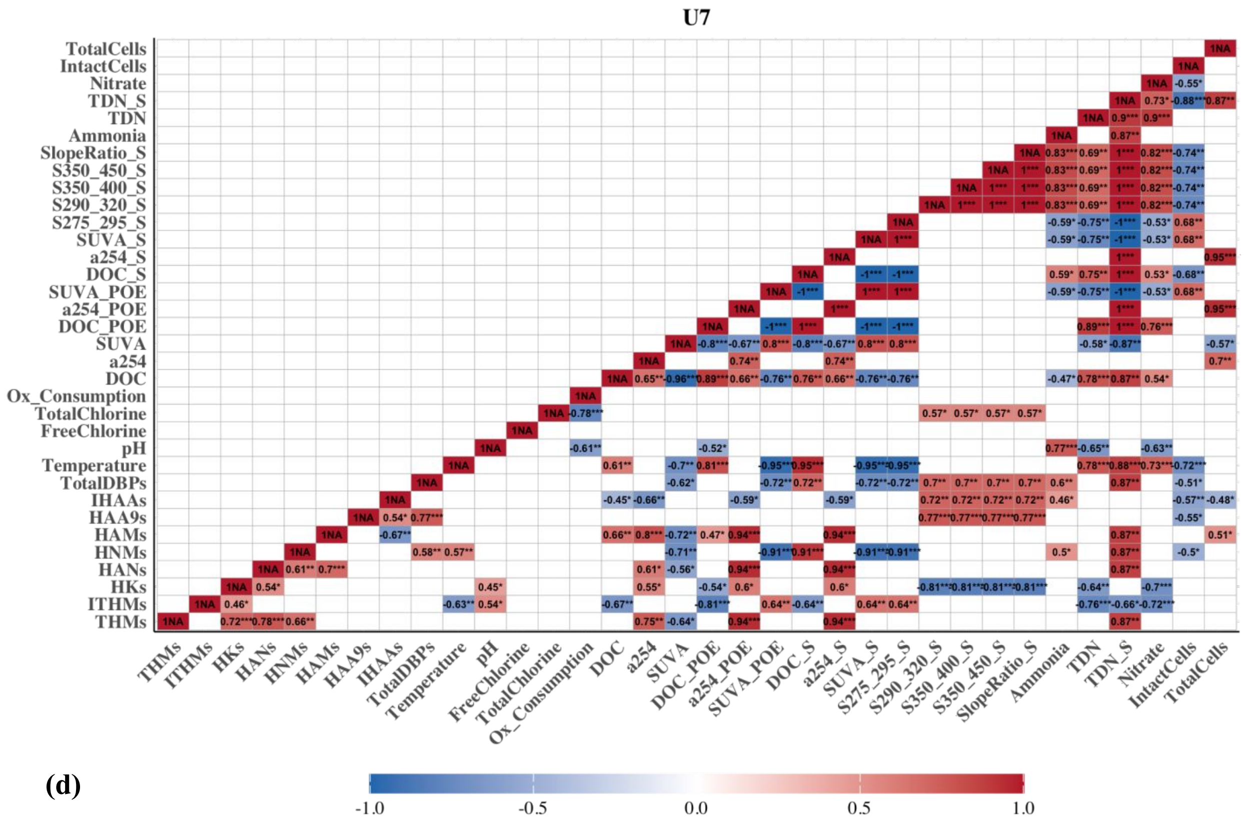
U7
TotalCells
IntactCells
Nitrate
TDN_S
TDN
Ammonia
SlopeRatio_S
S350_450_S
S350_400_S
S290_320_S
S275_295_S
SUVA_S
a254_S
DOC_S
SUVA_POE
a254_POE
DOC_POE
SUVA
a254
DOC
Ox_Consumption
TotalChlorine
FreeChlorine
pH
Temperature
TotalDBPs
IHAAs
HAA9s
HAMs
HNMs
HANs
HKs
ITHMs
THMs
-1.0
-0.5
0.0
0.5
1.0

(d)

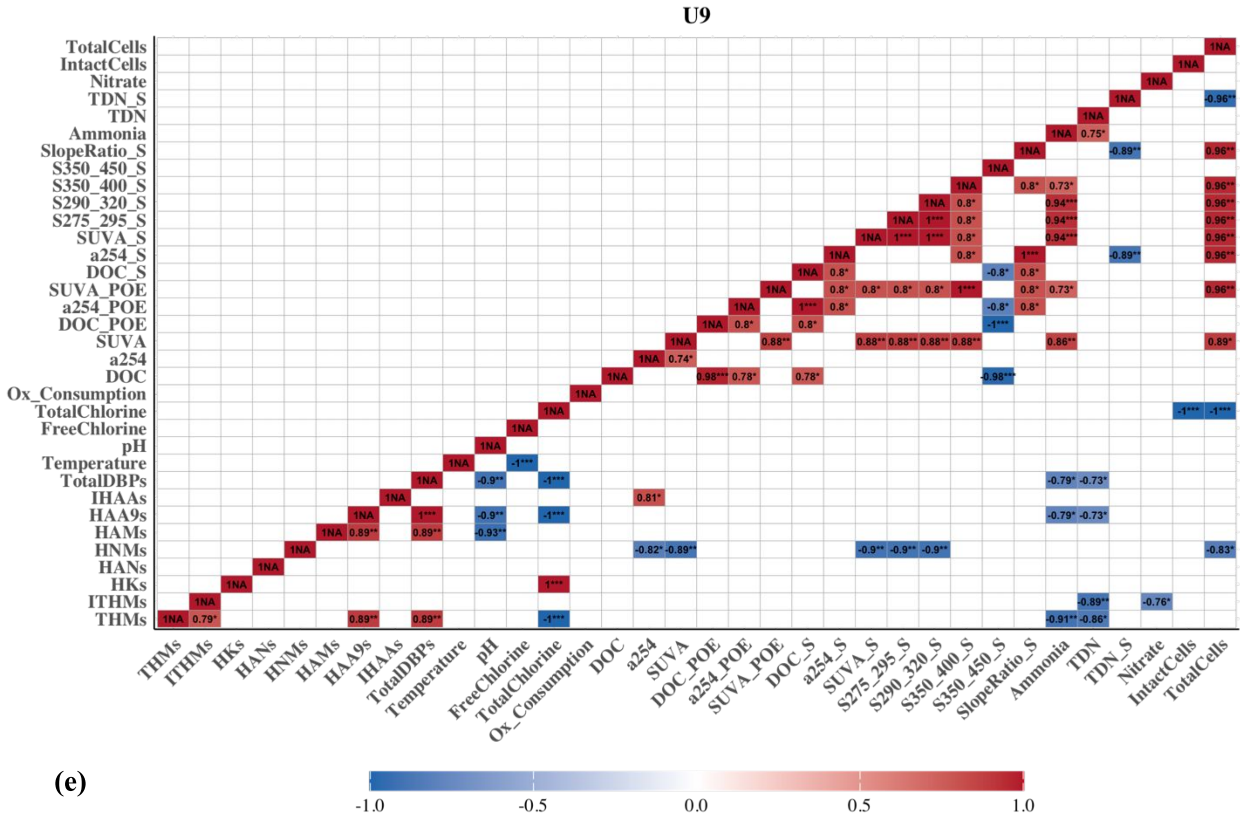

(e)

Figure SB5. Spearman correlations for DBP concentrations, water quality parameters, and microbial cell counts at individual chloraminated utilities (a) U3, (b) U4, (c) U5, (d) U7, and (e) U9.

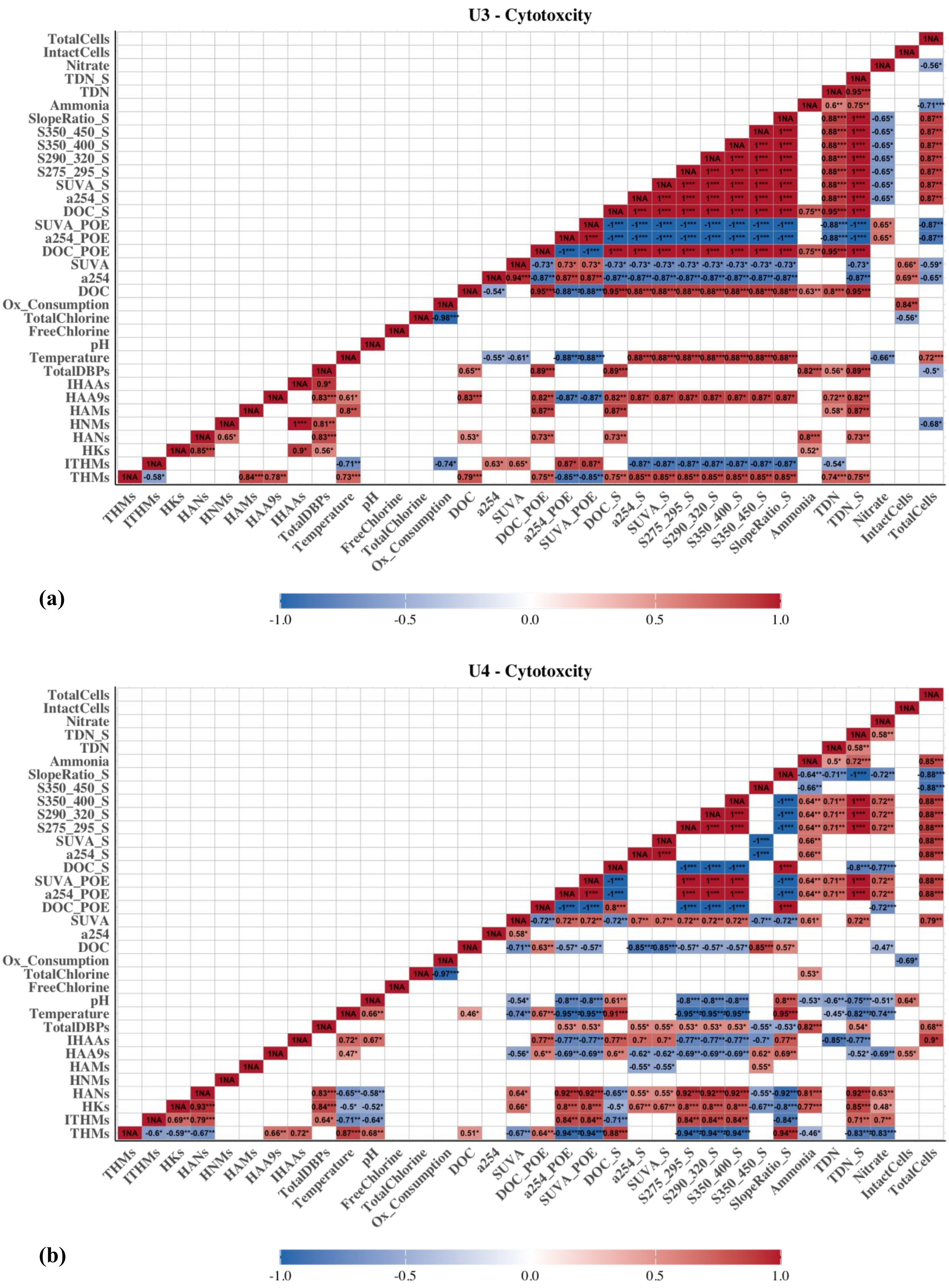
U3 - Cytotoxcity
TotalCells
IntactCells
Nitrate
TDN_S
TDN
Ammonia
SlopeRatio_S
S350_450_S
S350_400_S
S290_320_S
S275_295_S
SUVA_S
a254_S
DOC_S
SUVA_POE
a254_POE
DOC_POE
SUVA
a254
DOC
Ox_Consumption
TotalChlorine
FreeChlorine
pH
Temperature
TotalDBPs
IHAAs
HAA9s
HAMs
HNMs
HANs
HKs
ITHMs
THMs
(a)
-1.0
-0.5
0.0
0.5
1.0
U4 - Cytotoxcity
(b)
-1.0
-0.5
0.0
0.5
1.0

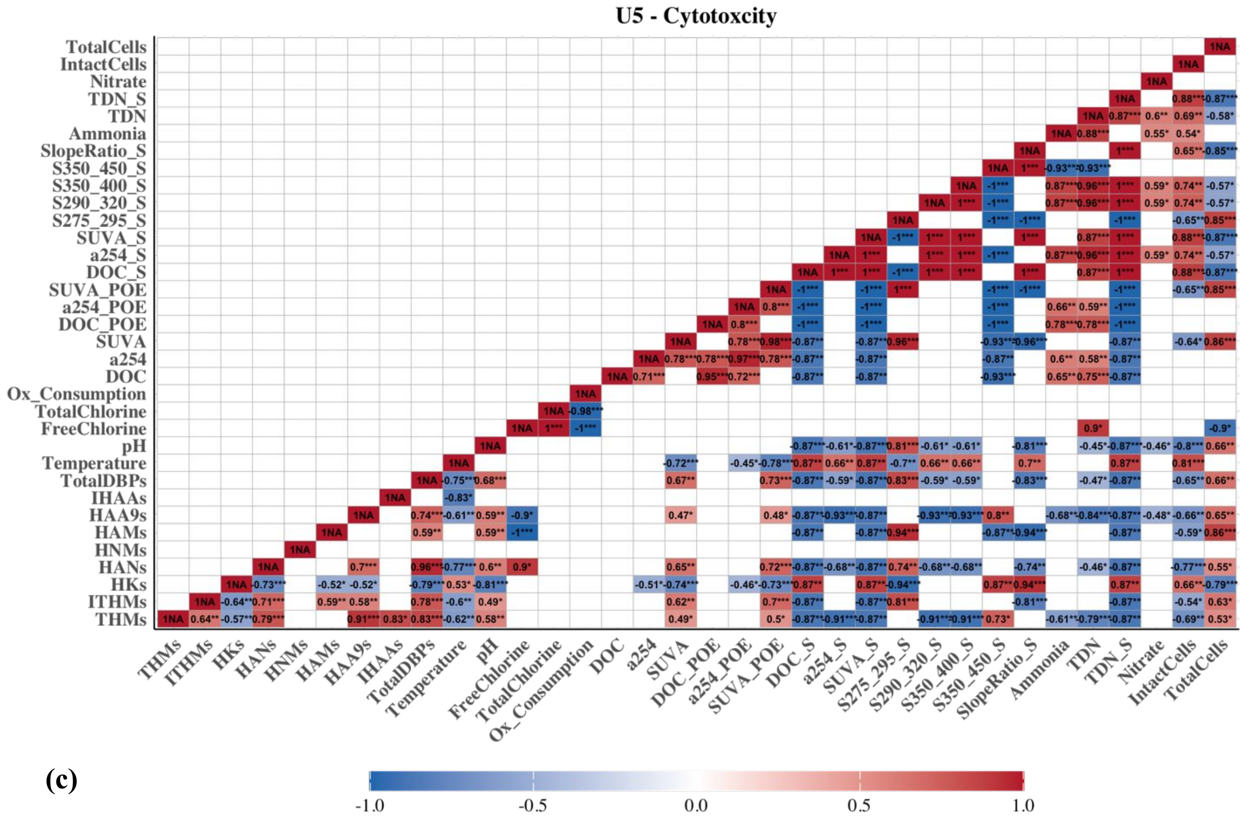
U5 - Cytotoxcity
TotalCells
IntactCells
Nitrate
TDN_S
TDN
Ammonia
SlopeRatio_S
S350_450_S
S350_400_S
S290_320_S
S275_295_S
SUVA_S
a254_S
DOC_S
SUVA_POE
a254_POE
DOC_POE
SUVA
a254
DOC
Ox_Consumption
TotalChlorine
FreeChlorine
pH
Temperature
TotalDBPs
IHAAs
HAA9s
HAMs
HNMs
HANs
HKs
ITHMs
THMs
-1.0
-0.5
0.0
0.5
1.0

(c)

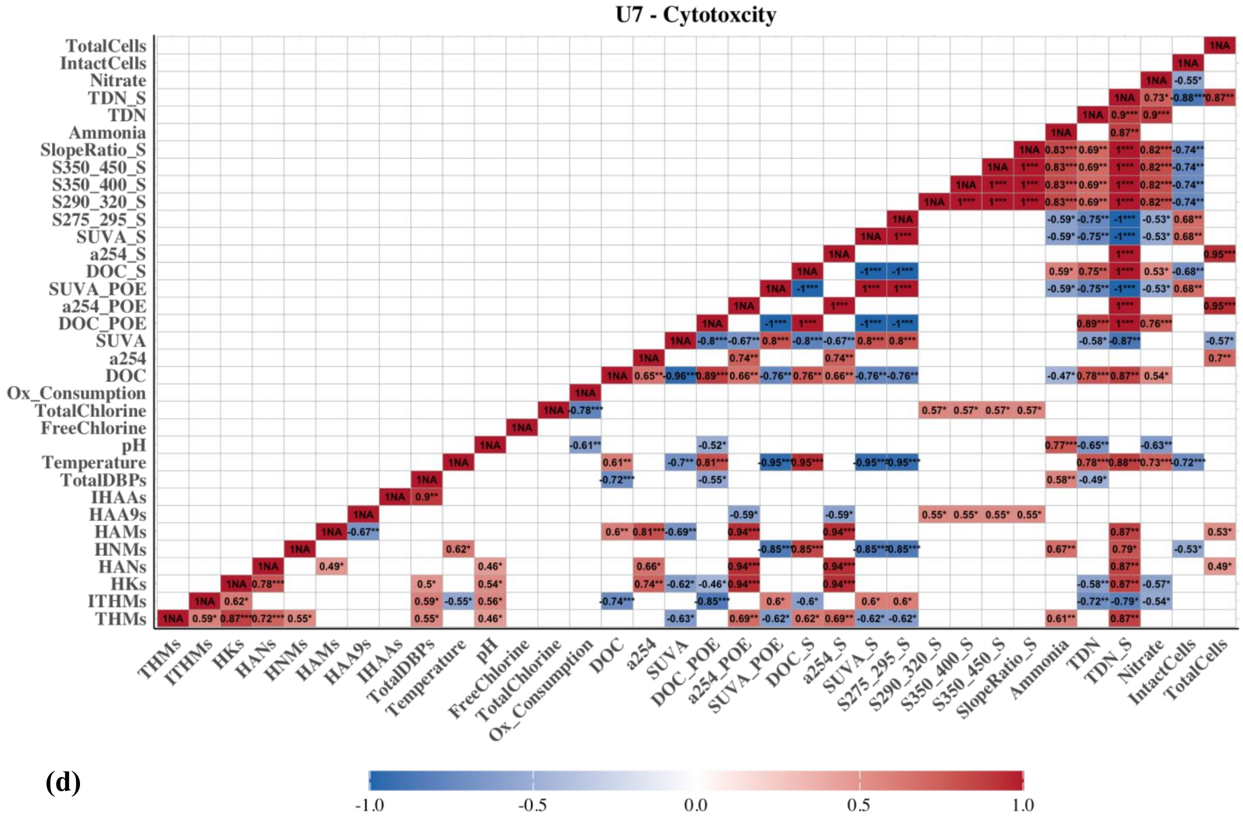
U7 - Cytotoxcity
TotalCells
IntactCells
Nitrate
TDN_S
TDN
Ammonia
SlopeRatio_S
S350_450_S
S350_400_S
S290_320_S
S275_295_S
SUVA_S
a254_S
DOC_S
SUVA_POE
a254_POE
DOC_POE
SUVA
a254
DOC
Ox_Consumption
TotalChlorine
FreeChlorine
pH
Temperature
TotalDBPs
IHAAs
HAA9s
HAMs
HNMs
HANs
HKs
ITHMs
THMs
-1.0
-0.5
0.0
0.5
1.0

(d)

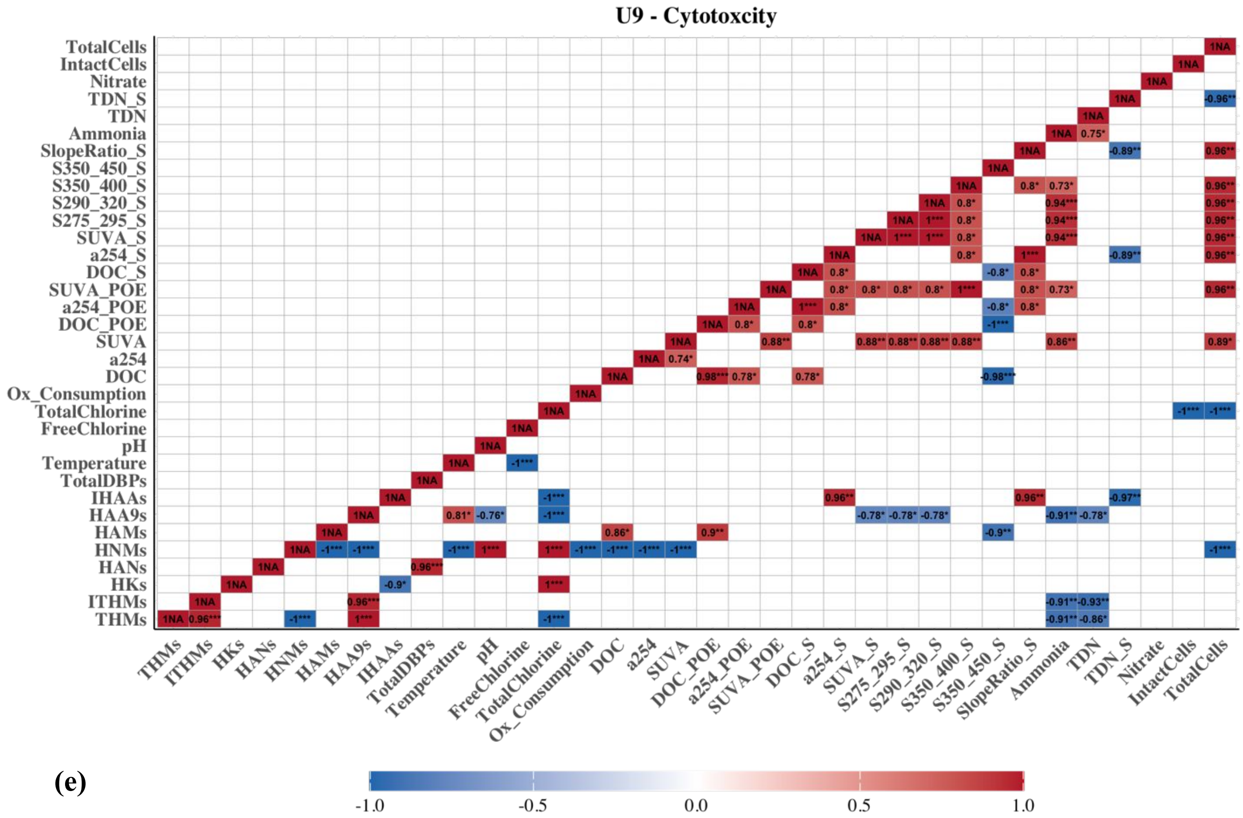

Figure SB6. Spearman correlations for DBP cytotoxicity, water quality parameters, and microbial cell counts at individual chloraminated utilities (a) U3, (b) U4, (c) U5, (d) U7, and (e) U9.
